# An integrative approach to bilingual cognition: preliminary insights into phonetic learning and sensorimotor adaptation

**DOI:** 10.3389/fnhum.2025.1549435

**Published:** 2025-07-25

**Authors:** Laura Spinu, Yasaman Rafat, A. Duke Shereen, Bradley P. Sutton, Maida Percival, Anastasiia Myslyk, Jiyoon Kim

**Affiliations:** ^1^Department of Communications and Performing Arts, Kingsborough Community College, City University of New York, Brooklyn, NY, United States; ^2^Graduate Center, Speech-Language-Hearing Sciences, City University of New York, New York, NY, United States; ^3^Department of Languages and Cultures, Western University, London, ON, Canada; ^4^Advanced Science Research Center at the Graduate Center, Neuroscience Initiative, City University of New York, New York, NY, United States; ^5^Department of Bioengineering, Grainger College of Engineering and Beckman Institute, University of Illinois at Urbana-Champaign, Urbana, IL, United States; ^6^Phonetics Laboratory, Faculty of Linguistics, Philology, and Phonetics, University of Oxford, Oxford, United Kingdom; ^7^Graduate Program in Speech-Language Pathology, Touro University, Brooklyn, NY, United States

**Keywords:** articulatory skill, auditory sensory memory, serial memory, proficiency, phonetic and phonological learning, cognitive effects of bilingualism

## Abstract

**Introduction:**

This study investigates the cognitive consequences of bilingualism by examining phonetic learning, speech motor adaptation, and verbal memory.

**Methods:**

Early Spanish-English bilinguals divided into high and intermediate proficiency groups and English monolinguals completed three tasks: (1) production of an artificial English accent with novel phonotactic rules, (2) serial digit span in English, and (3) production of unfamiliar speech sounds during real-time magnetic resonance imaging (rtMRI).

**Results:**

Bilinguals, particularly those with high proficiency, outperformed monolinguals in phonetic and articulatory learning. In the memory task, no group-level differences emerged overall, but high bilinguals showed stronger primacy effects at moderate sequence lengths, suggesting more efficient encoding.

**Discussion:**

These results support a shift toward investigating task-specific and process-based effects of language experience. We also demonstrate the feasibility of using rtMRI to assess articulatory behavior in cognitive studies of bilingualism, with minimal need for manual post-processing.

## 1 Introduction

The field of bilingual cognition is at an impasse. For years, the literature has reported the existence of a bilingual cognitive advantage (Bialystok et al., 2012; Antoniou et al., 2015). More specifically, bilingual language experience has been associated with some form of cognitive enhancement, such as improved cognitive control or protection against age-related cognitive decline. The bilingual advantage, however, has proven to be controversial due to consistent difficulties in replicating relevant results (Marzecová, 2015). Research involving bilingualism has mostly abstracted away from the complexities posed by the fact that, on the one hand, language experience is not a straightforward, easily measurable concept and, on the other hand, the effects of language knowledge on (neuro)cognition are similar to, and difficult to distinguish from the effects of other cognitive activities e.g. playing musical instruments or practicing mental exercises such as chess or sudoku. It is not unusual to find words such as “need”, “plea”, or “appeal” among recent titles: *Clear theories are needed to interpret differences: Perspectives on the bilingual advantage debate* (de Bruin et al., 2021), *Bilingualism and Cognitive Reserve: A Critical Overview and a Plea for Methodological Innovations* (Calvo et al., 2014), or *A Bilingual Advantage? An Appeal for a Change in Perspective and Recommendations for Future Research* (Poarch and Krott, 2019). All of these articles, among others, recognize the critical need for innovative approaches and recommend moving beyond the false dichotomy of “advantage vs. no advantage” when evaluating how bilingualism shapes cognition (Antoniou et al., 2023). This shift calls for focused attention to previously understudied domains through which bilingualism may shape cognition, particularly those involving the sensorimotor underpinnings of language. We position our approach in line with these calls for granularity, focusing on specific sensorimotor and learning domains rather than a binary advantage debate.

### 1.1 Behind the controversy

Several reasons, both methodological and conceptual in nature, have been invoked as potentially underlying the conflicting bilingual cognition findings (Valian, 2015). These include individual differences such as talent (Obler and Fein, 1988), language-pair factors (Higby et al., 2013), the fact that the bilingual advantage might be most apparent in childhood and old age but muted in adulthood (Bialystok et al., 2012), the existence of non-linguistic ways in which cognitive function can be improved e.g. musical expertise (Peretz and Zatorre, 2005; Zuk, 2014), and experimental task complexity across studies (Antoniou et al., 2015). Among these factors, the lack of a good operational definition of bilingualism and the omission of lower-level, sensorimotor functions in considering the relationship between language and cognition have begun to receive more systematic attention in recent literature.

#### 1.1.1 Lack of a standardized measure for bilingual language experience and over-reliance on self-reports

Language knowledge is subject to various lifestyle circumstances (Masgoret and Gardner, 2003), including speaker-internal, speaker-external, and interlanguage properties (e.g. diversity and intensity of language use, age of acquisition, amount of social interaction in each language, typological distance between a bilingual speaker's two languages, etc.). Many of these can, and have been, quantified (Marian et al., 2007), albeit this type of information is generally based on subjective self-reports, the validity of which can vary considerably across skills, proficiency levels, and raters (Marian and Hayakawa, 2021). Recent studies have stressed that not all bilinguals are the same and have pleaded for more detailed assessments of bilingual experience (de Bruin, 2019). In our study, we adopt this perspective and acknowledge that bilingual experience exists along a continuum. Variability in bilingual profiles is not just noise, but a feature that can significantly affect cognitive outcomes; this recognition is one of the steps toward resolving the current replication difficulties in the field (Antoniou et al., 2023).

Few studies have employed objective measures of language knowledge (Gollan et al., 2012; Lemhöfer and Broersma, 2012), which remain incomplete (often addressing just one aspect of language knowledge, such as vocabulary size or verbal fluency). DeLuca et al. (2019) stand out by employing a standardized proficiency test, the Oxford Quick Placement Test (Geranpayeh, 2003), a test of English for ESL speakers. Numerous authors have ascribed the bilingual cognition controversy to the field's general over-reliance on self-reports, often recommending the use of standardized language proficiency testing in future experimental paradigms (Marian and Hayakawa, 2021; de Bruin, 2019). Crucially, these recommendations are supported by recent literature on the neurobiology of bilingualism (Del Maschio and Abutalebi, 2018; DeLuca et al., 2019) and the neural convergence hypothesis Green (2003) suggesting that proficiency, rather than age of acquisition, may be the critical differential factor in the functional organization of bilingual language processing. In parallel with methodological critiques, there has been a growing recognition of the need to expand the range of cognitive domains being investigated.

#### 1.1.2 Shifting focus: sensorimotor and memory-based effects of bilingualism

Much of the earlier work on the cognitive effects of bilingualism focused on executive function, based on the assumption that bilinguals constantly manage cross-linguistic activation and must resolve linguistic conflict during processing (Poarch and Krott, 2019; Marian and Spivey, 2003; Thierry and Wu, 2007). It has been proposed that this control mechanism engages domain-general cognitive mechanisms associated with executive function (Luk, 2011), including working memory, cognitive flexibility, and inhibitory control.^1^ A number of studies reported bilingual advantages in tasks tapping these processes (Green and Abutalebi, 2013), though many other studies have questioned the robustness and generalizability of such effects.

While executive function has dominated the bilingualism literature, other cognitive domains (including sensorimotor processes associated with language) have been relatively overlooked. Articulatory control, auditory-motor integration, and somatic memory play central roles in language use and acquisition (Simmonds et al., 2011; Kröger, 2011), yet the impact of bilingualism on these lower-level mechanisms has only recently begun to receive empirical attention. Simmonds et al. (2011) emphasized the need to investigate sensorimotor learning in bilinguals more than a decade ago, but progress in this area has remained limited.

Recent studies have started to fill this gap. Bilinguals have been shown to outperform monolinguals in tasks involving phonetic and phonological learning, articulatory flexibility, and auditory sensory memory (Dugaillard and Spinu, 2019; Spinu, 2023). According to the cognitive permeation hypothesis (Lindenberger et al., 2000), sensorimotor tasks increasingly recruit cognitive resources with age, implying that experience, such as bilingual language use, can modulate sensorimotor performance. Schäfer et al. (2006) argue that sensorimotor and cognitive systems are causally intertwined, especially under varying cognitive load. This suggests that sensorimotor domains may provide an alternative lens for observing bilingual effects, one that complements rather than depends on executive function frameworks. The present study is grounded in this broader perspective. We focus on phonetic learning, articulatory control, and serial memory. In addition to cognitive-behavioral assessments, we incorporate rtMRI to visualize articulatory gestures with high spatial and temporal resolution. This method allows semi-automatic extraction of articulator dynamics, enhancing the ecological validity and precision of our measures. Although perception is not assessed directly, all learning tasks involve auditory exposure to novel accents or sounds, making perceptual encoding an integral, if implicit, part of the learning process. These domains bridge linguistic, sensorimotor, and higher-level cognitive systems. Our primary aim is to explore how bilingual experience shapes these domain-specific capacities, particularly those rooted in auditory and articulatory processing, which remain underexamined despite their core contribution to language use.

### 1.2 Phonetic and phonological learning: a more robust cognitive difference between monolinguals and bilinguals?

Designing controlled experiments that incorporate the full complexity of bilingual experience is challenging. Nevertheless, recent studies suggest bilinguals, regardless of their specific linguistic backgrounds, show superior learning in phonetic and phonological domains. A study examining vocabulary learning with foreign phonetic contrasts (Antoniou et al., 2015) revealed a bilingual advantage, which was influenced by the universal difficulty of the specific contrast and the phonetic similarity between the target language and the learners' native language (Kopečková, 2016). Additionally, research involving non-native contrasts (Tremblay and Sabourin, 2012) suggests that multilinguals and bilinguals possess enhanced speech perception abilities compared to monolinguals, even when initial discrimination abilities are similar across groups.

Adding to these perceptual studies, recent work has also assessed production-based phonetic and phonological learning in bilinguals. In Spinu et al. (2018), the performance of 17 monolinguals was compared with 25 bilinguals in a production experiment involving two tasks: imitation and spontaneous reproduction of a novel foreign accent, Sussex English (spoken in SE England). The focus was on a sound already present in the participants' speech inventory, the glottal stop [ʔ], but mapped differently in the novel accent. Bilinguals demonstrated superior learning, exhibiting a significantly greater increase in [ʔ] usage post-training, despite both groups displaying similar immediate imitation performance. This led the authors to speculate that auditory sensory memory strategies, potentially underpinned by stronger subcortical sound encoding in bilinguals (Krizman et al., 2012), may facilitate the remapping between existing mental sound representations and articulatory command configurations.

In a subsequent study (Spinu et al., 2023), 31 monolingual and 31 early bilingual speakers were trained on a novel artificial accent that differed from standard North American English in four specific ways. The bilingual group consistently outperformed the monolingual group on all four linguistic features, both during training (consisting of direct imitation of the sentences containing the novel features) and testing phases. This advantage was particularly evident in the testing phase, where bilinguals demonstrated enhanced ability to combine multiple features. Monolinguals, on the other hand, reverted to baseline performance when required to produce sentences with all four features, suggesting an effect of task complexity (Valian, 2015; Antoniou et al., 2015). The same study showed a bilingual advantage in auditory sensory memory. Bilinguals outperformed monolinguals on a suffixed digit span task, especially as the task's complexity increased. The authors speculated that bilinguals possess longer auditory sensory memory span, which was found to correlate strongly with performance on the phonetic and phonological learning task. This indicates that sensory functions such as auditory sensory memory may play a part in novel accent learning. This line of research has expanded our understanding of the cognitive differences associated with bilingualism while raising questions about the underlying mechanisms involved in phonetic and phonological learning. The consistency of the learning advantage across diverse bilingual backgrounds suggests the presence of a core bilingual trait that is less affected by external variability. Identifying this domain and its underlying mechanisms can clarify which aspects of bilingualism drive robust cognitive differences, providing a more reliable foundation for future research than broader domains, where variability in language experience and background often obscures effects.

The findings reported above lay the groundwork for the present study, which seeks to gain insight into the mechanisms behind bilinguals' learning strategies through multimodal assessment. We examine articulatory learning and sensorimotor processes in bilinguals, using rtMRI to capture fine-grained articulatory behavior. Although the current study does not primarily target executive function, it contributes to ongoing debates about the cognitive consequences of bilingualism. Our serial memory task probes verbal working memory, which is often considered a component of executive function. Primacy effects observed in digit span tasks have long been associated with working memory capacity, whereas recency effects implicate auditory sensory memory. By examining both early and late list positions in our analysis, we can have a closer look at how bilingual experience interacts with these separable memory systems. Our primary focus, however, is on the sensorimotor foundations of phonetic learning and auditory memory. As discussed, these domains are less frequently examined in bilingualism research, yet may offer more consistent and interpretable effects. In this way, our study bridges sensorimotor, linguistic, and executive domains, broadening the scope beyond traditional executive function accounts.

The inclusion of rtMRI in our study was motivated by its ability to capture dynamic, mid-sagittal views of multiple articulators during naturalistic speech production. Unlike other articulatory methods such as ultrasound, electromagnetic articulography (EMA), or electropalatography (EPG), rtMRI provides a broader field of view that includes the tongue, velum, pharynx, and laryngeal structures, allowing researchers to observe how individual articulators are configured during speech without the need for invasive instrumentation or extensive calibration. For bilingual speakers, who often exhibit cross-linguistic influence in articulatory habits, rtMRI enables the identification of articulator-specific patterns that may differ systematically depending on language background or learning stage. In this preliminary study, the goal was not to analyze inter-articulator coordination *per se*, but rather to use rtMRI as a high-resolution tool to assess how individual articulators are shaped during the production of novel sounds in a second language. Because rtMRI requires minimal post-processing and allows for continuous data collection during connected speech, it was particularly well-suited to capturing the subtle articulatory differences that emerge as speakers attempt to produce unfamiliar phonetic targets. Future work may expand on these findings by leveraging rtMRI's full potential to analyze coordination patterns across articulators, which would offer further insight into the sensorimotor adjustments that underlie phonetic learning.

## 2 The present study

The main goal of the present study is to address some of the challenges described in the previous sections by focusing on the integration of sensorimotor perspectives into the examination of the cognitive impacts of bilingualism. We explore differences in phonetic and phonological learning, as well as auditory sensory memory and articulatory skill, between monolinguals and early bilinguals of varying proficiency levels. The inclusion of an articulatory component is innovative and opens a new research direction in the bilingual cognition literature. Our main research questions are whether group differences exist in:

1. Phonetic and phonological learning (specifically, the ability to learn a new accent of a language they already speak).
2. Sensorimotor function:

a. Articulatory skill (as reflected by the learning of novel speech sounds)
b. Auditory sensory memory (as reflected by performance on the final items of digit span task with suffix effect)

Lastly, in light of recent proposals that proficiency, rather than age of acquisition, may be the critical differential factor in the functional organization of bilingual language processing (Del Maschio and Abutalebi, 2018; DeLuca et al., 2019), we also explored how L2 proficiency levels might influence these outcomes, although our relatively small sample size prevents us from drawing statistical conclusions.

## 3 Materials and methods

Drawing on previous work (Spinu, 2023; Spinu et al., 2018, 2023), the current study includes an experiment with several tasks, as detailed in the following subsections.

### 3.1 Participants and procedure

Twenty adults (7 males and 13 females) participated in the study. The mean age of participants was 23.8 years (range 18–53, SD = 7.5). Of these, 10 identified as English-Spanish early bilinguals and 10 as monolingual speakers of English. All participants signed an informed consent form approved by the CUNY Institutional Review Board and were compensated for approximately 2 hours of participation. Each participant completed a detailed language background questionnaire. Based on their answers, two groups were identified: 10 bilinguals and 10 monolinguals. Among the bilinguals, the mean age was 24.8 years (SD = 10.44, median = 22), and the group consisted of 8 females and 2 males. Among the monolinguals, the mean age was 22.9 years (SD = 3.75, median = 22.5), with an even split of 5 females and 5 males.

All of the bilingual participants reported acquiring Spanish before age 2. English exposure began at birth for three participants, at ages 1–2 for five for them, and at 3–4 years for the remaining two. They all considered themselves (near-)native in both languages. They all identified with Spanish-speaking cultures (8 of them listed these as their primary cultural identification while the remaining 2 listed them as a secondary identification after US/American culture). All bilinguals reported having regular exposure to and use of both Spanish and English in daily life. An online Spanish proficiency test confirmed that their abilities spanned intermediate to high levels, with scores between 42% to 90%. Participants' English proficiency was not formally assessed, as all participants were living and studying/working in New York City, and they had all been raised in the U.S., with English reported as being used at least 50% of the time. Based on this context, we assumed high functional proficiency in English across both groups.

Of the monolingual participants, eight listed only English as a known language. The remaining two listed minimal passive exposure (Yiddish, ASL) but reported 95-100% English usage. Based on these descriptions, we consider them functionally monolingual, acknowledging Grosjean's perspective that monolingualism exists on a continuum Grosjean (2024).

Upon arrival at the lab, participants received a brief overview of the experiment and had the opportunity to ask questions before proceeding. They then undertook a series of tasks in a fixed order. The bilingual participants completed an additional language proficiency assessment in Spanish as their last task, but otherwise all participants followed the same procedure. In total, participants performed three main experimental tasks: (1) a phonetic and phonological learning task involving training on a novel accent, (2) a serial memory task (forward digit span with a suffix), and (3) a novel sound learning task involving articulatory imaging with rtMRI. Participants were informed about the nature of each task (e.g., digit span, novel accent learning, unfamiliar sound production) and provided a short overview of each before beginning. These verbal instructions were reinforced by clear written task instructions displayed on-screen within the experimental software at the start of each task, and participants were encouraged to ask for clarification at any time.

### 3.2 Informed consent and background questionnaire

The questionnaire administered prior to the lab visit was adapted from the Language Experience and Proficiency Questionnaire (LEAP-Q, Marian et al., 2007) and administered via the Qualtrics platform. The questionnaire was administered in English (no Spanish version was offered), as all participants lived, studied, or worked in New York City and had communicated exclusively in English with the experimenters up to that point. Participants were also invited to ask for clarification if any question was unclear. Among others, participants provided information about their age, gender, main caregivers' native language, their own language use and self-perceived proficiency in reading, speaking, understanding spoken and written language, past and current exposure to each language (if speakers of more than one language), order of acquisition, and whether they had hearing or speech problems. The data collected subsequently enabled us to determine the characteristics of interest for each of the participants, placing them into either a monolingual or bilingual group.

### 3.3 Phonetic and phonological learning of a new accent

For the phonetic/phonological learning task, we adapted the paradigm of Spinu et al. (2018, 2020, 2023) to train participants on a novel accent of English (artificially constructed). The participants were informed that the recordings reflected an artificial English accent, their task being to learn this accent to the best of their ability, and that they should attempt to imitate it during training for this purpose. This accent differed from standard New York City English in two features (for time considerations, we only selected two out of four features used in the original study). The novel accent features were:

1. Tapping: intervocalic /l/ → [ɾ] e.g. “color” → [kʌɾɚ]
2. Diphthongization: the vowel /ɛ/ → [jɛ] after an onset consonant, e.g. “bed” → [bjɛd]

It should be noted that the sounds involved exist in the English^2^ inventory (typically realized in different contexts), therefore their phonetic realization was not expected to pose challenges to either speaker group. The stimuli consisted of short sentences containing either one single feature, e.g. for tapping “This salad tastes good”. where the [l] in the word *salad* is pronounced as a tap, and for diphthongization “My head is pounding” where the vowel in *head* is diphthongized, or both features combined (epenthesis and diphthongization), as in “A funnier fellow I could not imagine” where the word *fellow* is a target for both diphthongization of its first vowel and tapping of the intervocalic [l] sound. We constructed a list of 15 sentences: 10 sentences with a single novel accent feature (5 tapping, 5 diphthongization), and 5 sentences with both features. All stimulus sentences were recorded in the artificial accent by a trained phonetician (female, native Northeastern U.S. English speaker). For reference, she also recorded the sentences in her natural accent, but participants only heard the artificial version during training. The recordings were checked manually in Praat (Boersma and Weenink, 1992–2025) to ensure that the target features were produced consistently in the artificial accent.

The novel accent learning task, presented via the online platform Pavlovia, consisted of three phases: baseline, training, and testing. In the baseline phase, each participant was recorded reading 15 sentences that contained the target features, spoken in their own natural accent). In the training phase, participants listened via headphones to 15 training sentences (different from the baseline/test sentences) spoken in the novel accent by the model speaker, and were then asked to imitate the same sentences. During the first part of training, they heard each sentence once in random order without any orthographic input. In the second part of training, each sentence was played again in random order, and participants were instructed to repeat it aloud immediately, imitating the novel accent as closely as possible. During this imitation phase, an orthographic transcription of each sentence was provided on the screen along with the audio. This design was intentional: presenting the written sentence during imitation ensured that all participants had equal access to the lexical content and could more precisely focus on reproducing the phonetic and phonological properties of the novel accent. It also maintained consistency with prior studies using the same or similar materials (Spinu et al., 2018, 2023), allowing for cross-study comparability.^3^ No information was provided regarding which features of the accent they should focus on. Each training sentence was presented twice in this manner (listen and imitate). During the testing phase, participants saw the original 15 baseline sentences on the screen in written form and were asked to reproduce them in the novel accent, this time without hearing any audio prompts. All productions were audio-recorded. Baseline and test productions were later analyzed to assess how well participants learned the accent (i.e., whether they incorporated the tapping and diphthongization features).

### 3.4 Serial memory

The digit span task was administered via Psyscope (Cohen et al., 1993). The digit string list was randomly generated in blocks of increasing length, starting with 3-digit sequences and ending with 10-digit sequences. Random strings included digits 1–9, each of which was generated only once per sequence, except for the 10-digit sequences where one of the digits was repeated. The English audio sequences were played over headphones, following which participants were asked to type in the digits in the order they had heard them using the computer keyboard. After each sequence, the program played the word “recall”, serving as a suffix. The role of the suffix is to disrupt auditory sensory memory processing, resulting in a diminished recency effect (Bloom, 2006; Li et al., 2013; Nees, 2016—see Footnote 1). Each of the blocks had 5 trials, following which the program moved to the next sequence length (whereby an additional digit would be included). Once each participant completed 40 trials (5 items x 8 sequence lengths, from 3 to 10 digits), the experimental task ended.

### 3.5 Novel sound learning task

#### 3.5.1 Stimuli and presentation

In the novel sound learning task, we investigated participants' ability to learn and produce unfamiliar speech sounds while images of their articulators were recorded using rtMRI. This task focused on three novel speech sounds that do not occur in English or Spanish. The sounds were chosen to engage different articulators in novel ways: a rounded high front vowel (lips), a palatalized labiodental consonant (tongue tip), and a nasalized vowel (velum). The selected sounds represent distinct articulatory challenges involving lip rounding, palatalization, and nasalization, allowing us to probe learning across different motor subsystems. Participants received brief training for each sound and were then tested on their production of those sounds. The speakers who produced the target words recorded 3 repetitions of each, and the second repetition was selected for presentation to the participants. The three novel sounds and their characteristics (with additional details and the full list of stimuli in Supplementary Appendix A) are as follows:

1. **Round high front vowel**: This vowel is articulated with the tongue high and front (as for English [i] as in “tea”), but with lip rounding (as in English [u] e.g. “too”). It occurs in languages such as French (e.g., tu [ty]) and Turkish. To train and test this sound, we selected English C(C)VC words containing the vowel [i] in a context where rounding could be applied (e.g., “keen”, “keel”, “steal”, “teen”). To avoid coarticulatory effects, none of the consonants was rounded. A female French-English bilingual (from Quebec) recorded each target word in the carrier sentence “I am reading the word.... presently,” pronouncing the target vowel as [y].
2. **Secondarily palatalized voiceless labiodental fricative**: This is a consonant produced like [f] (lower lip against upper teeth) while simultaneously raising the tongue toward the hard palate (as for English [j] in “you”). The addition of palatalization also results in more spreading of the lips. It is present in languages like Russian and Romanian. For training, we used English words ending in “-f” (CVC words such as “tough”, “hoof”, “staff”), which a female Romanian-English bilingual recorded in the carrier sentence with a palatalized [f^j^] at the end.
3. **Low back nasal vowel**: This vowel is produced with the velum lowered to allow airflow to pass through the nasal cavity, while the tongue is low and back as in [ɑ]. Phonemically nasalized vowels (and their oral counterparts) are encountered in French and other languages. For this target, we chose English words of the form C(C)VC containing the vowel “o” (as in “pop”, “stop”, “glob”). The same French-English bilingual who recorded the [y] stimuli also recorded these words in the carrier sentence, pronouncing the vowel with nasalization, i.e. [~ɑ].

While inside the MRI scanner, participants were first presented via the Pavlovia online platform with 48 words (16 per novel sound) in random order, which they were asked to read as they normally would in order to create a baseline set. Next they were trained and tested on each novel sound separately. The training procedure for the novel sounds mirrored that of the accent task described in Section 3.3: participants first listened to all the sentences (with the novel sound produced by the model) and, after the full set was played once, they listened to each sentence again and immediately repeated it, imitating the novel sound to their best ability. There were 16 training sentences for each sound, and each was heard twice and imitated once. During the imitation part of the training, participants saw the sentences in English orthography on a screen. Even though pronounced in a novel way, the orthography for the target words remained as in English, e.g. [tyl] was spelled “teal”, consistent with the instructions received, which specified they would be learning a novel accent of English.

The participants were then tested by being presented with one word at a time (without a carrier sentence) and being asked to read it three times in a row, trying their best to replicate the accent they had just been trained on. While the words including the three different sounds of interest were mixed in baseline (for a total of 48 words presented in random order), for training and testing of each novel sound participants were presented with all 16 words pertaining to that sound randomized in separate blocks. The order of the three sounds was the same for all participants: round high front vowel, palatalized labiodental fricative, and nasal low back vowel.

#### 3.5.2 MRI acquisition

Images of the participants' articulators were acquired during the baseline block, as well as the testing block of the three novel sounds, but not during training (listening and imitation), to allow for quiet learning conditions without being distracted by the MRI noises. We used a Siemens 3 Tesla Prisma MRI Scanner equipped with a 20-channel head coil. After completing a standard safety screening form for magnetic resonance procedures, the participants were screened for ferromagnetic materials using a handheld detector and then accompanied into the room housing the MRI scanner, where a research assistant ensured they were feeling comfortable after lying supine onto the patient table (with a pillow), and provided them with further explanations on the procedure. Images of the participants' articulator movements were acquired in four separate runs, for baseline and for the testing of the three novel sounds, as detailed below. This imaging component allowed us to directly observe whether bilinguals and monolinguals differ in how they adapt articulatory gestures when learning new sounds.

Participants spoke into a noise-attenuating MR-compatible optical microphone Dual Channel-FOMRI III (Optoacoustics). This acoustic signal was later used for manual segmentation of the target words in Praat (Boersma and Weenink, 1992–2025). Because the MR system gradients introduced a high level of noise during the recording, we did not perform acoustic analysis of the recordings made within the MR scanner as their quality was not good enough for spectral analysis. Initial scans included a localizer and a static T2-weighted Turbo Spin Echo (TSE) scan. The localizer is a short scan used to determine the orientation for the imaging sequences. The T2-weighted TSE scan produced a high-definition 3D image of the vocal tract to assist with manual slice placement for MRI scans.

Dynamic MRI scans were acquired using a customized gradient echo FLASH MRI pulse sequence (Jin et al., 2023). Image acquisitions included a temporal navigator acquisition interleaved with an imaging data acquisition, reconstructed with a low rank approach to yield a very high frame rate of approximately 101 frames per second. The FLASH sequence had TR/TE = 4.940/2.0 ms, flip angle of 10, spatial resolution of 1.875x1.875x8 mm3, for a single midsagittal slice. The alignment between audio and images was accomplished through a TTL pulse output from the sequence that was recorded with the microphone.

### 3.6 Proficiency test

The Spanish proficiency test administered to the bilingual participants was selected to include different components (grammar, vocabulary, and reading comprehension) and be relatively short (a total of 50 questions), so as not to extend the total duration of the experiment by too long. This test was administered last so that the order of the experimental tasks was identical for both participant groups (monolingual and bilingual) up to that point. At the time of testing, the test was freely available online at the https://www.transparent.com/learn-spanish/proficiency-test web page.

### 3.7 Analyses

#### 3.7.1 Phonetic and phonological learning of a new accent

Categorical judgments of whether a feature was present or not were provided by a bilingual graduate student (Spanish-English), raised in a Spanish-speaking household in the U.S., who received training, and verified by the first author. Each target feature (diphthongization and tapping) was scored with a 1 if present and 0 if absent. The judgments were based on spectrographic evidence and any unclear cases were double-checked by the first author. A mean accent score was computed for each participant for the baseline, training (imitation), and testing blocks. The statistical analysis was a univariate ANOVA comparing the groups' performance across the different blocks and features.

#### 3.7.2 Serial memory

Serial memory performance was analyzed using two complementary accuracy metrics: whole-trial accuracy and per-digit accuracy. Whole-trial accuracy was defined as the proportion of trials in which all digits were recalled in the correct serial order. Per-digit accuracy was defined as the proportion of individual digits recalled in the correct position, averaged across all valid trials (i.e., those where the number of recalled digits matched the input sequence length). Accuracy was computed for each participant and sequence length. Linear mixed-effects (LME) models were conducted separately for each accuracy metric. These models included fixed effects for Group (Monolingual, Intermediate Bilingual, High Bilingual), Sequence Length (3–10 digits), and their interaction (participant as random effect). In addition, serial position effects were examined by comparing accuracy for the first two (primacy) and last two (recency) positions in each sequence. Paired t-tests were used to compare primacy and recency performance within each group and sequence length, and one-way ANOVAs were used to assess group differences in primacy-recency difference scores. Tukey HSD *post hoc* tests were performed where applicable.

#### 3.7.3 Novel sound learning task

**Figure 1:**
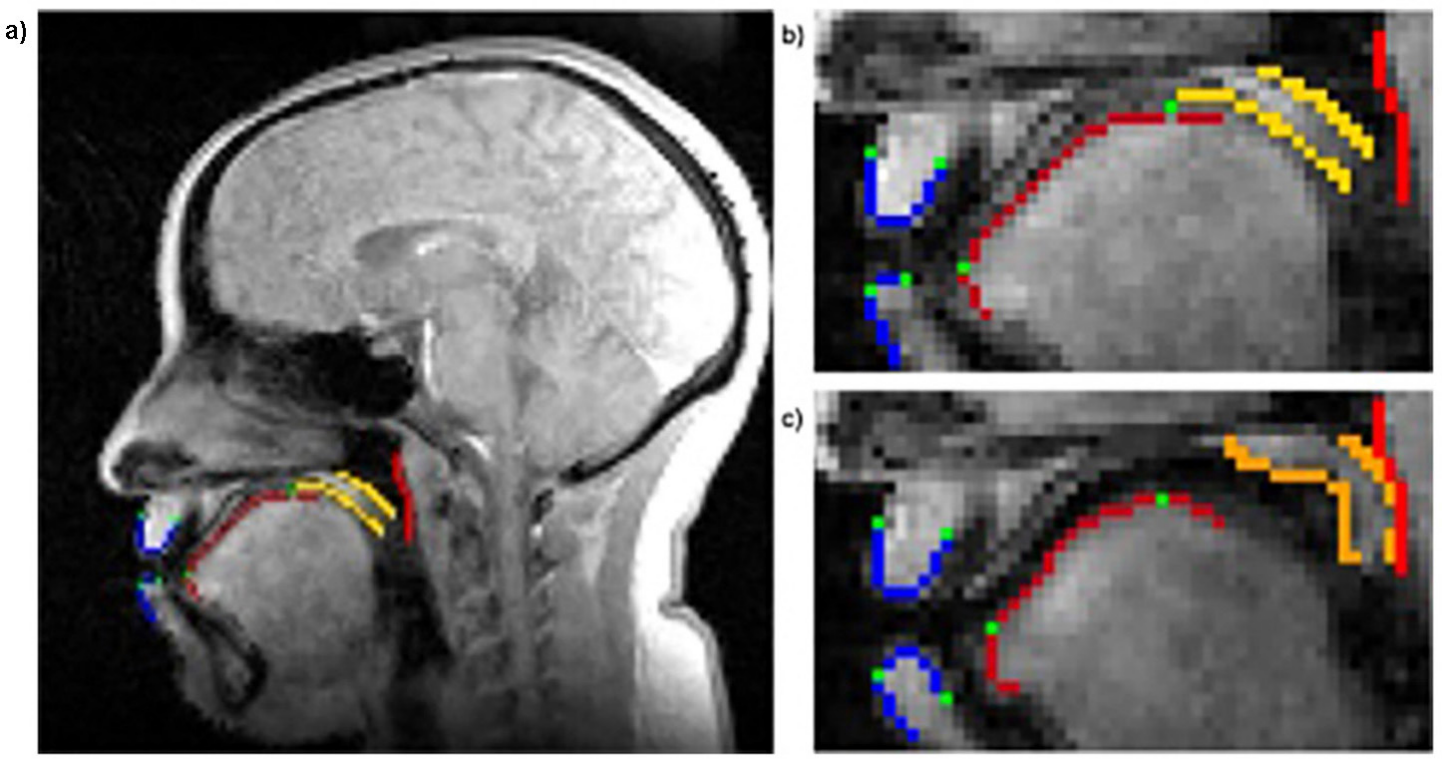
Articulator tracking outcome, with the green points representing the core coordinates used for statistical analysis: lower lip tip, lower lip wall, upper lip tip, upper lip wall, tongue tip, tongue dorsum. The posterior wall is tracked in red. **(a)** Mid-sagittal MRI image of the vocal tract showing traced contours of key articulators during speech. Velum interaction can be visualized when the velum pixels turn orange **(c)** from yellow **(b)**.

The data were reconstructed using a low-rank reconstruction method (rank 40), where a temporal basis function constructed from the navigator was employed to estimate the spatial basis (Jin et al., 2023; Fu et al., 2017). Using the reconstructed images, post-processing was performed with a custom semi-automated tracking code developed in MATLAB R2024a. The workflow includes a manual step where users are required to select anatomical regions of interest, including the tongue, velum, upper and lower lip movements, the posterior wall, the region of overlap between the velum and tongue, and the overlap between the hard palate and tongue (see Figure 1). After manual selection, the automated phase of the code utilizes the edgelink function (https://www.peterkovesi.com/matlabfns/LineSegments/example/index.html) to label major edges in the images by connecting points into edge lines for streamlined data processing. Extracted features were then exported into an Excel file, with speech data matched to the corresponding image frames to facilitate statistical analysis.

Articulator position was recorded for each frame, throughout the duration of each word. The articulators considered were the tongue tip, tongue dorsum, lower lip tip, upper lip tip, lower lip wall, upper lip wall, and velum. Except for the velum measure, which tracked contact between the velum and the posterior wall (as a measure of nasalization), each articulator had two values, in the horizontal (fronting) and vertical (height) plane. These values are denoted with 1 (x axis, horizontal) and 2 (y axis, vertical) in what follows. Articulatory displacement (in mm) between baseline and testing was calculated for each articulator and sound. Group-level comparisons were performed using univariate ANOVAs for each articulator-sound combination, with Speaker Type (Bilingual, Monolingual) as the between-subjects factor. For the velum, which was assessed categorically (presence/absence of closure), group comparisons were based on mean percent velum contact during nasalized vowel production. The results were summarized descriptively and visually, with additional interpretation of effect sizes and individual variation patterns.

#### 3.7.4 Proficiency test

The Spanish proficiency test we employed was made up of four parts addressing different aspects of linguistic competence: grammar 1 (select the correct option), grammar 2 (select the word that is incorrect), vocabulary, and reading comprehension. Upon completion, the website provided a single composite score over the four different parts (out of 50). Each bilingual participant received a score which was later used in the correlation analyses.

### 3.8 Results

In this section, we report results from the proficiency test and each of the three main tasks: the phonetic/phonological learning task, the serial memory task, and the articulatory learning task using MRI. We conclude with a section exploring correlations among these measures.

#### 3.8.1 Proficiency test

Because Spanish proficiency scores were used to subdivide our 10 bilingual participants into two groups for subsequent analyses, we report them first. The scores obtained ranged from 21/50 (42%) to 45/50 (90%). The specific scores were: 21/50, 24/50, 32/50, 32/50, 34/50, 35/50, 39/50, 41/50, 43/50, and 45/50. The mean score was 34.6/50 (69.2%), the median was 34.5/50 (69%), and the standard deviation was approximately 7.5 points (15%). Based on a median split – a common approach for identifying meaningful contrasts in small samples – five participants scored in the **intermediate** proficiency range ( ≤ 34/50), while five participants reached the **high** proficiency range (>34/50). The two lowest scores were obtained by two male participants who had only had one Spanish-speaking main caregiver, with the second caregiver speaking English natively. Both speakers had been raised in the US. While one of them described his use of Spanish as limited to interacting with family (both as he grew up and currently), the other reported more varied Spanish use in both situations, specifically through interacting with family (10/10), interacting with friends (5/10), reading (6/10), internet use (6/10), watching TV (6/10), and listening to music (7/10), where 0 means “never” and 10 means “all the time”. This variation in proficiency reflects a realistic distribution of bilingual experiences in an urban U.S. setting (New York City). The eligibility requirements as described in our recruitment materials were for participants to have been speaking both English and Spanish regularly since before age 3, and be fluent (native or near-native) in both of these languages. The wide range of scores obtained is surprising in light of this, highlighting the need for a more systematic approach to recruiting and categorizing bilinguals as discussed in Section 1.1.1.

#### 3.8.2 Phonetic and phonological learning of a new accent

This section reports how well participants learned to produce features of a novel accent. Participants (Monolinguals, Intermediate(-proficiency) bilinguals, and High(-proficiency) bilinguals) were trained to imitate two unfamiliar speech features (tapping and diphthongization) embedded in English sentences. A trained rater judged whether the features were produced appropriately in context, yielding a categorical accuracy score.

**Figure 2:**
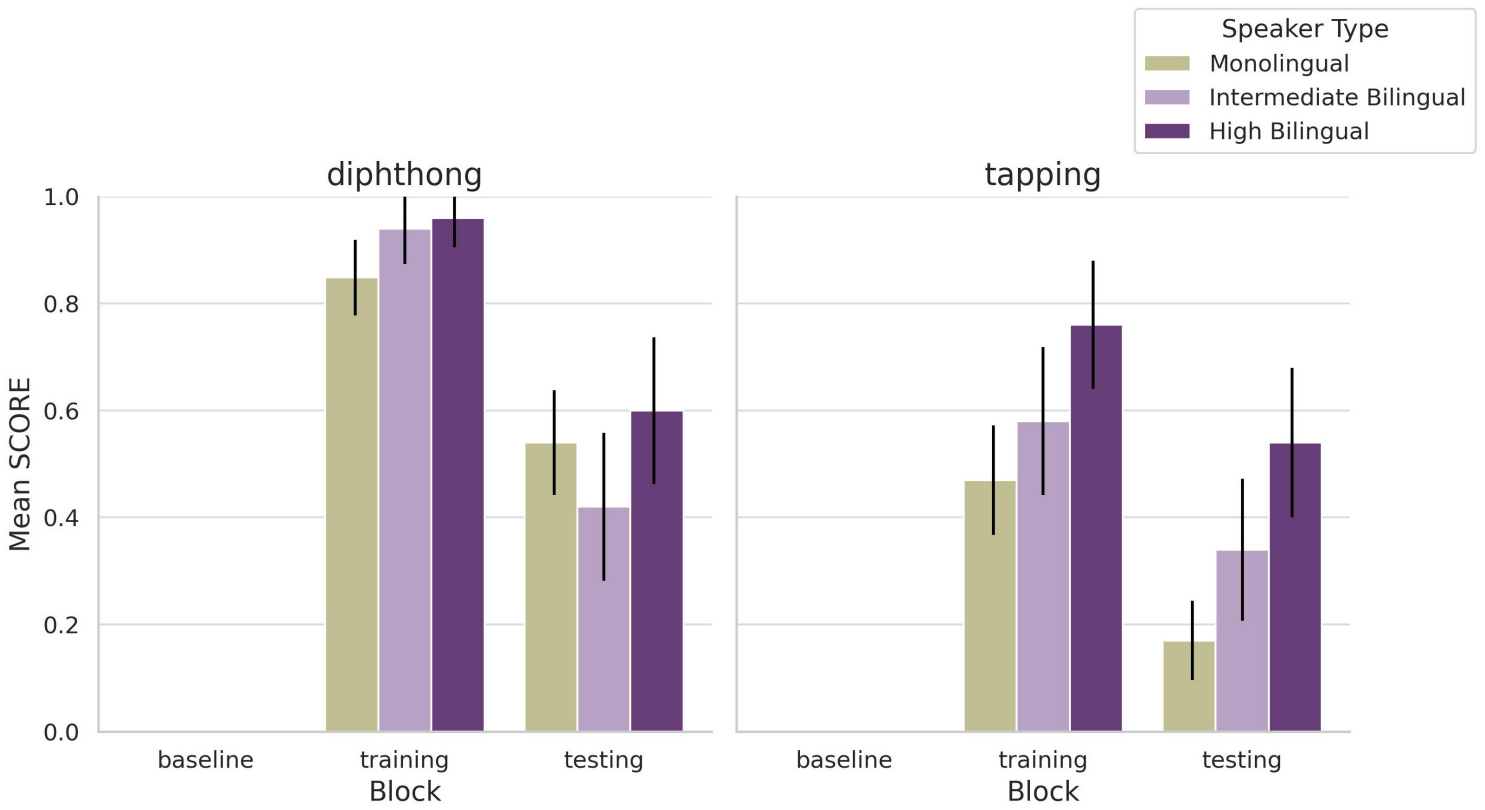
Mean of accent scores for each of the novel accent features obtained by monolinguals and the two bilingual groups in baseline, training and testing. As expected, the new features did not exist in either group's production in baseline. Error bars represent the standard error of the mean.

Figure 2 shows mean performance for each speaker group across three blocks (baseline, training, and testing) for each feature. As expected, no group spontaneously produced the features during baseline. All groups improved significantly with training, as confirmed by a three-way ANOVA with accent score as the dependent variable and speaker group, block, and feature as factors. There were significant main effects of speaker group: *F*_(2, 1180)_ = 14.88, *p* < 0.001; block: *F*_(2, 1180)_ = 428.90, *p* < 0.001; and feature: *F*_(1, 1180)_ = 77.94, *p* < 0.001. All two-way interactions were also significant: speaker group × block: *F*_(4, 1180)_ = 3.91, *p* = 0.004; speaker group × feature: *F*_(2, 1180)_ = 5.66, *p* = 0.004; and block × feature: *F*_(2, 1180)_ = 21.58, *p* < 0.001. The three-way interaction among speaker group, block, and feature was also significant: *F*_(4, 1180)_ = 2.62, *p* = 0.034.

*Post hoc* comparisons using the Bonferroni correction revealed that both bilingual groups outperformed monolinguals during training for both features (*p* < 0.01). The high-proficiency bilinguals showed the highest scores during training and retained their advantage during testing for the tapping feature, outperforming both monolinguals and intermediate bilinguals (*p* < 0.05). For diphthongization during testing, no significant differences were observed between groups. These results suggest that bilingual experience may offer an advantage in phonetic learning, and that greater proficiency is associated with more robust learning and retention, especially for phonological rules like tapping. However, bilingual performance was not uniform across features, consistent with previous findings of feature- and task-specific effects.

#### 3.8.3 Serial memory

**Figure 3:**
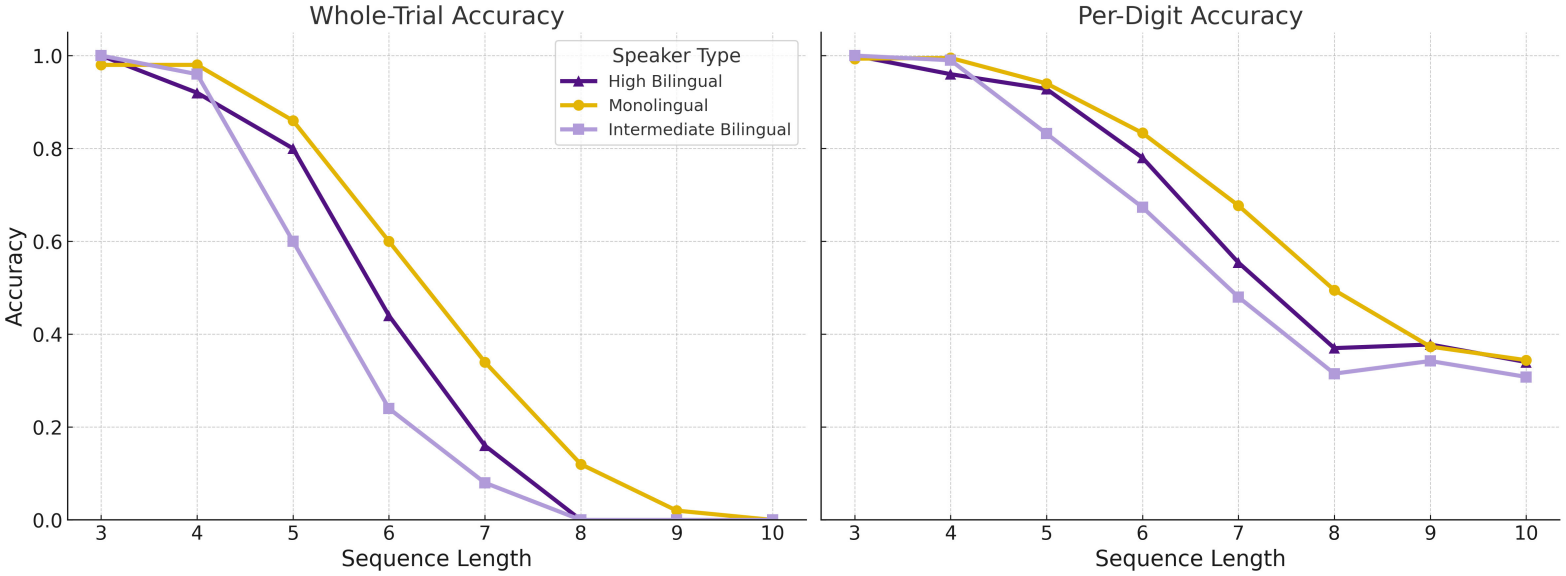
Accuracy by sequence length across speaker groups. **Left**: Whole-trial accuracy, calculated as the proportion of trials where all digits were recalled in the correct serial order. **Right**: Per-digit accuracy, calculated as the proportion of digits recalled in the correct position, averaged across all trials (excluding trials where the number of digits recalled did not match the input length). Accuracy declined with increasing sequence length, but no significant group differences were observed for either metric. Error bars are omitted for clarity.

Figure 3 presents the effects of sequence length on memory performance, measured through whole-trial and per-digit accuracy, across the three groups. These data were analyzed as described in what follows. A linear mixed-effects (LME) model was conducted separately for whole-trial and per-digit accuracy, with Group (Monolingual, High Bilingual, Intermediate Bilingual) and Sequence Length (3–10 digits) as factors. For both accuracy types, there was a robust main effect of Sequence Length (β = −0.104, *p* < 0.001), indicating that accuracy declined as sequence length increased. However, no significant main effect of Group was found for either whole-trial (*F*_(2, 114)_ = 1.54, *p* = 0.219) or per-digit accuracy (*F*_(2, 114)_ = 0.66, *p* = 0.518). There was also no significant Group × Sequence Length interaction for either measure. These results suggest that while memory performance declines reliably with sequence length, there were no statistically significant differences in overall performance between the three speaker groups.

**Figure 4:**
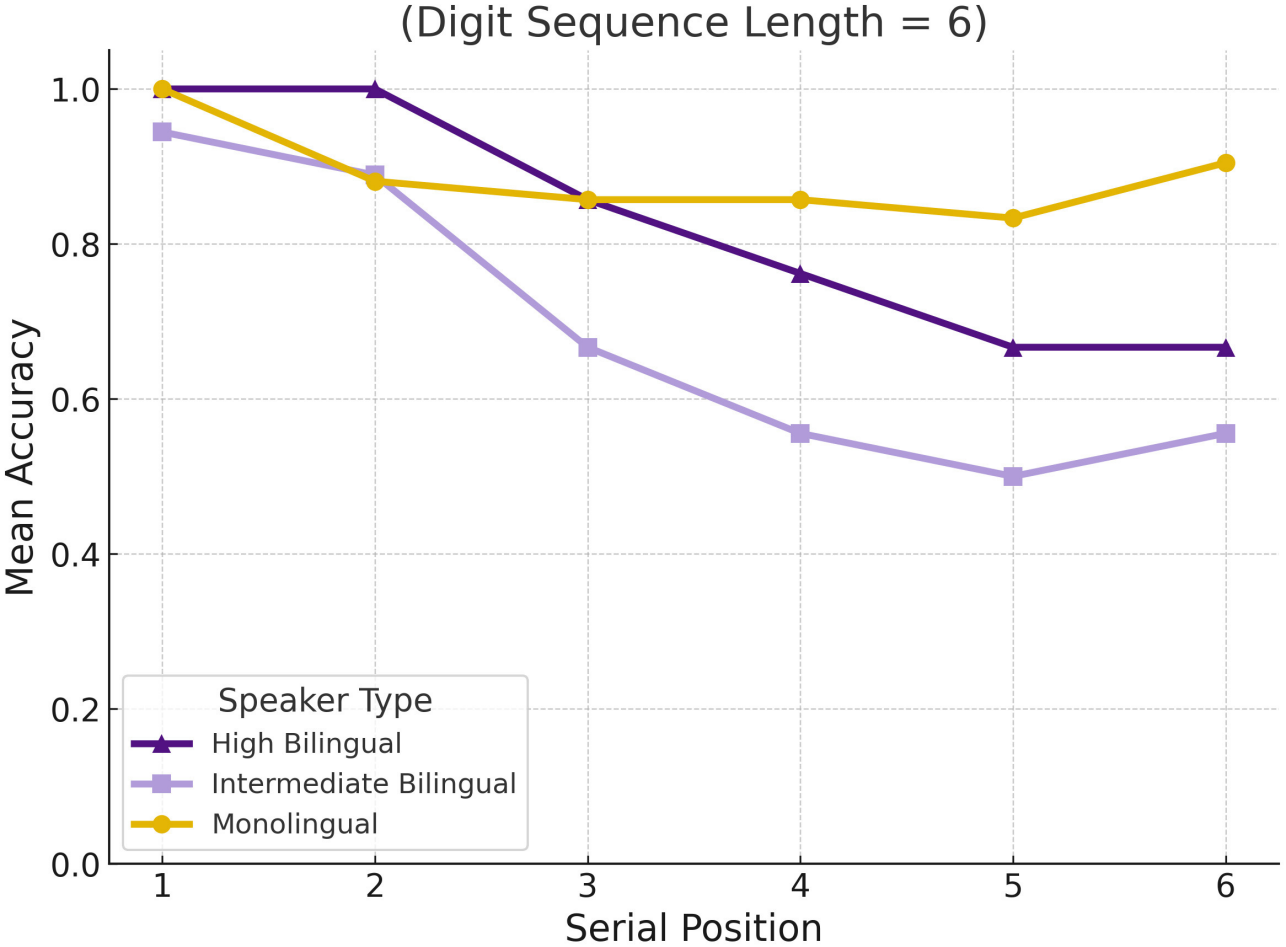
Mean accuracy by group for sequence length 6. Circles = Monolinguals; Triangles = High Bilinguals; Squares = Intermediate Bilinguals.

To examine differences in serial position effects across groups, we calculated the mean difference in accuracy between primacy (first two digits) and recency (last two digits) positions for each participant and sequence length. A one-way ANOVA on these difference scores revealed a significant group effect at sequence length 6, *F*_(2, 16)_ = 11.23, *p* = 0.0008, but not at other lengths. Tukey *post hoc* tests showed that High Bilinguals exhibited significantly greater primacy effects than both Intermediate Bilinguals (mean difference = 0.406, *p* = 0.002) and Monolinguals (mean difference = 0.273, *p* = 0.036). Intermediate Bilinguals and Monolinguals did not differ significantly from each other (mean difference = 0.134, *p* = 0.589). These findings suggest that High Bilinguals may engage more robust encoding or rehearsal strategies early in the sequence, particularly at moderate memory loads. Figure 4 illustrates group-level mean accuracy by serial position for digit span sequences of length six, revealing performance differences among monolingual, high bilingual, and intermediate bilingual participants.

#### 3.8.4 Novel sound learning task

This section presents the articulatory changes made by bilingual and monolingual participants when producing three unfamiliar sounds introduced during training. Due to technical issues preventing the analysis of all participants, we report here results based on data from 5 monolinguals and 5 bilinguals. Four of the bilinguals analyzed formed a relatively homogenous subset, with scores ranging from 32/50 (64%) to 35/50 (70%) and a mean of 33.25/50. The remaining bilingual had obtained the lowest proficiency score of the group, specifically 21/50. Consequently, we treated bilinguals as a single group, as further division into high and intermediate-proficiency groups was not statistically feasible as in previous sections. For one of the monolingual participants, poor image quality prevented the analysis of the nasal vowel testing run. The analysis is complete for all other participants.

An examination of individual articulatory changes across speaker groups and sounds reveals that bilinguals exhibit more systematic articulatory adaptations than monolinguals in the expected direction for successful acquisition (more lip movement for round vowels, slightly more tongue movement for palatalized labial, and less velum contact for nasal vowels). Bilinguals produce overall more lip movement and tongue fronting across all sounds, with one participant (P6) consistently demonstrating the most pronounced fronting changes in all conditions. In contrast, monolinguals display more variability, with some participants showing clear articulatory shifts (e.g., P10 lowering all articulators) while others remain relatively stable. These patterns tentatively suggest that bilinguals may develop more consistent articulatory strategies when adapting to novel sounds, whereas monolinguals demonstrate a broader range of individual differences. A detailed analysis and corresponding figures are included in the Supplementary Appendix B.

**Figure 5:**
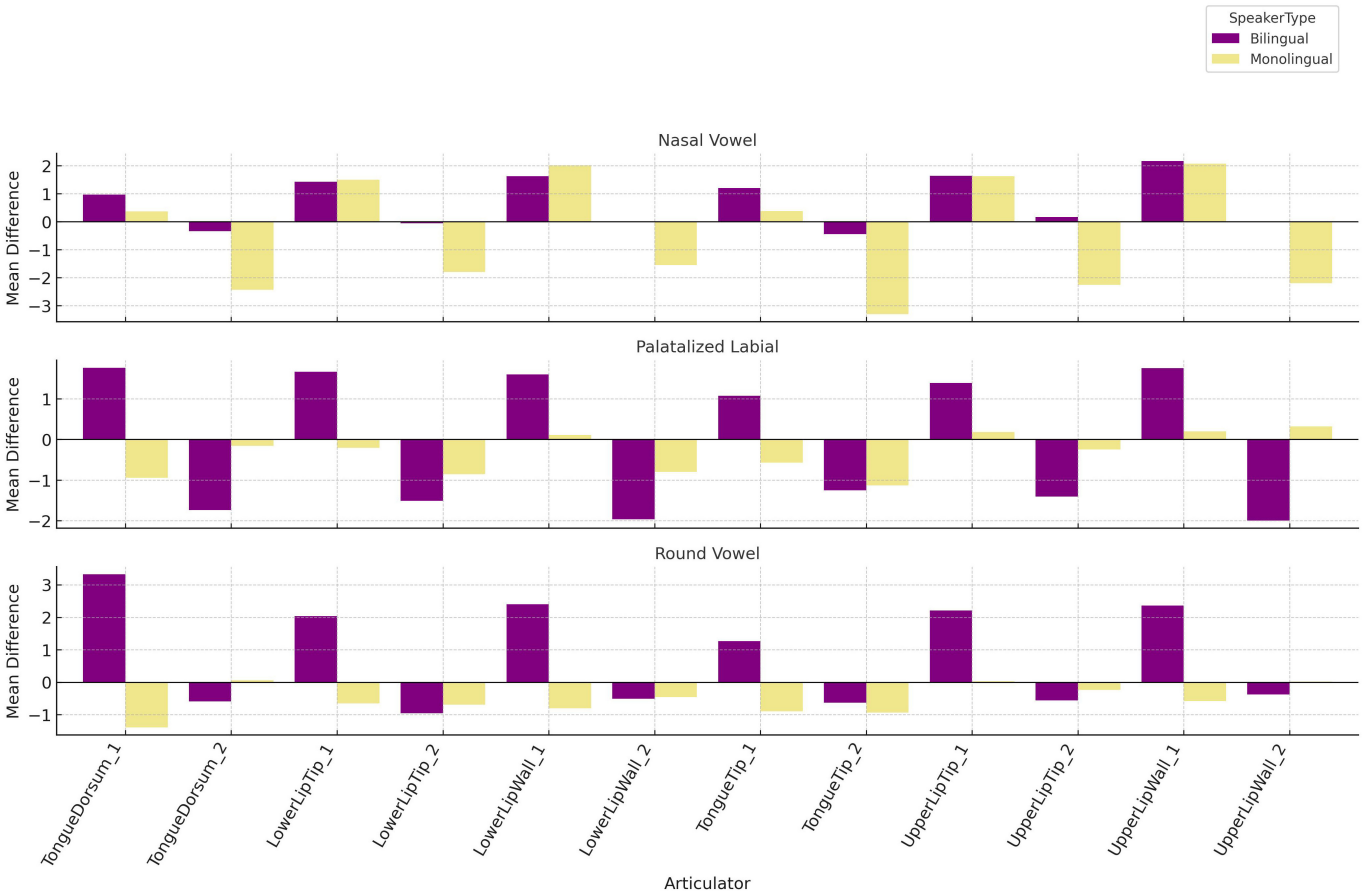
Mean difference (in mm) between conditions (testing–baseline) for the two groups.

Table 1 displays a summary of expected and observed articulatory changes for each of the three novel sounds. At the group level, bilinguals showed greater articulatory adjustments, suggesting more flexible speech motor learning. For each sound, we highlight the three articulators that changed the most for each group. ANOVA results (F-values, *p*-values, and η^2^) are provided below. These results are best interpreted in conjunction with Figure 5, which shows the magnitude of articulatory displacement for each articulator. *Note:* Velum contact is excluded from this analysis, as it was measured categorically (presence/absence of closure) rather than in millimeters of displacement.

1. Round vowel. Bilinguals showed the most movement in the tongue dorsum and lips:

- **Dorsum_1**: +3.32 mm, SpeakerType *F*_(1, 64, 341)_ = 10,847.2, *p* < 0.001, η^2^ = 0.144
- **LowerLipWall_1**: +2.4 mm, SpeakerType *F*_(1, 64, 341)_ = 6,744.394, *p* < 0.001, η^2^ = 0.095
- **UpperLipWall_1**: +2.36 mm, SpeakerType *F*_(1, 64, 341)_ = 7,587.812, *p* < 0.001, η^2^ = 0.105

**Table 1:** Summary of expected and observed articulatory changes for each novel sound. Bilinguals generally showed more systematic, larger, and target-aligned articulatory adaptations across sounds.

**Sound type**	**Expected change (for successful learning)**	**Observed: bilinguals**	**Observed: monolinguals**
Round vowel	Tongue tip same (high and front) or higher/more fronted if hyperarticulated; upper lip raised/fronted; lower lip lowered/fronted	Greater displacement in dorsum and lips (e.g., +3.32 mm dorsum); more systematic lip and tongue fronting	Less consistent articulatory change; lower displacement in dorsum (–1.39 mm) and tongue tip; more variability across participants
Palatalized labial fricative	Tongue tip higher/more fronted; lips more spread; upper lip raised, lower lip backed	Greater lip and dorsum displacement (e.g., +1.76 mm dorsum, –2 mm upper lip wall); moderate tongue involvement	Smaller changes; focused on tongue tip (−1.13 mm) and dorsum; less cohesive than bilinguals
Nasalized vowel	Decreased velum contact; tongue body same or slightly lowered	Largest changes in upper/lower lip articulators (+2.16 mm upper lip wall); 12% nasalization increase	More tongue involvement (e.g., dorsum −0.95 mm); smaller nasalization change (6%)

Monolinguals also showed most displacement in the dorsum but relied more on the tongue tip:

- **Dorsum_1**: −1.39 mm, SpeakerType *F*_(1, 64, 341)_ = 10,847.2, *p* < 0.001, η^2^ = 0.144
- **Tip_2**: −0.94 mm, SpeakerType *F*_(1, 64, 341)_ = 2.85, *p* = 0.091, η^2^ = 0.000
- **Tip_1**: −0.9 mm, SpeakerType *F*_(1, 64, 341)_ = 8,439.25, *p* < 0.001, η^2^ = 0.116

2. Palatalized labial. Bilinguals made larger changes using both lips and the tongue dorsum:

- **UpperLipWall_2**: −2 mm, SpeakerType *F*_(1, 62, 069)_ = 80.734, *p* < 0.001, η^2^ = 0.001
- **LowerLipWall_2**: −1.97 mm, SpeakerType *F*_(1, 62, 069)_ = 110.603, *p* < 0.001, η^2^ = 0.002
- **Dorsum_1**: +1.76 mm, SpeakerType *F*_(1, 62, 069)_ = 18,218.044, *p* < 0.001, η^2^ = 0.227

Monolinguals produced smaller changes, again involving the tongue tip and dorsum:

- **Tip_2**: −1.13 mm, SpeakerType *F*_(1, 62, 069)_ = 229.625, *p* < 0.001, η^2^ = 0.004
- **Dorsum_1**: −0.95 mm, SpeakerType *F*_(1, 62, 069)_ = 18,218.044, *p* < 0.001, η^2^ = 0.227
- **LowerLipTip_2**: −0.86 mm, SpeakerType *F*_(1, 62, 069)_ = 17.12, *p* < 0.001, η^2^ = 0.000

3. Nasal vowel. Bilinguals produced the largest changes in upper and lower lip articulators:

- **UpperLipWall_1**: +2.16 mm, SpeakerType *F*_(1, 58, 434)_ = 12,875.616, *p* < 0.001, η^2^ = 0.181
- **UpperLipTip_1**: +1.64 mm, SpeakerType *F*_(1, 58, 434)_ = 11,499.445, *p* < 0.001, η^2^ = 0.164
- **LowerLipWall_1**: +1.62 mm, SpeakerType *F*_(1, 58, 434)_ = 9,748.019, *p* < 0.001, η^2^ = 0.143

Monolinguals relied more on tongue tip and lip tip articulators:

- **Tip_2**: −1.13 mm, SpeakerType *F*_(1, 58, 434)_ = 251.567, *p* < 0.001, η^2^ = 0.004
- **Dorsum_2**: −0.95 mm, SpeakerType *F*_(1, 58, 434)_ = 502.128, *p* < 0.001, η^2^ = 0.009
- **UpperLipTip_2**: −0.86 mm, SpeakerType *F*_(1, 58, 434)_ = 529.7, *p* < 0.001, η^2^ = 0.009

**Figure 6:**
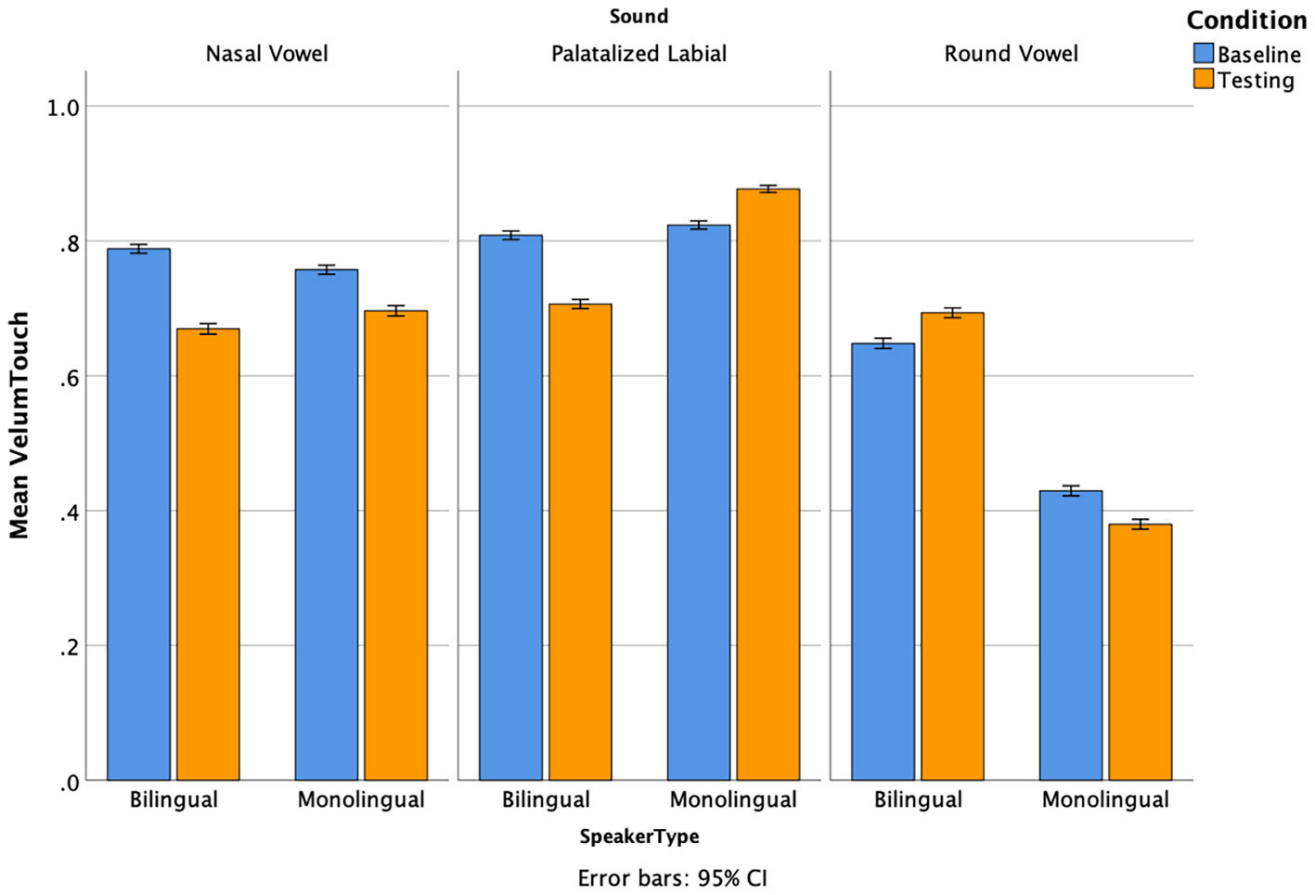
Mean velum touch by condition and speaker type for each sound. The higher the value, the less nasalization occurred as contact between the velum and the posterior wall blocked the airflow from entering the nasal cavity. More nasalization occurred when the values were lower.

Figure 6 displays nasalization percentages for the three sounds of interest in baseline and testing, broken down by speaker type. Velum Touch refers to the velum making contact with the posterior wall. Thus, higher values refer to lower levels of nasalization, as the airflow was prevented from entering the nasal cavity. Lower values indicate a higher degree of nasalization. While a certain amount of nasalization is expected to occur independently of novel sound production, the mean values should decrease for the nasal vowel block if this sound was acquired successfully. In our data, both groups exhibited high velum contact at baseline, around 80% in bilinguals and slightly less in monolinguals. Following training, both groups showed a reduction in velum contact for the nasal vowel, with bilinguals decreasing by approximately 12% and monolinguals by 6%. Although this numerical difference suggests potentially more consistent acquisition in bilinguals, statistical analyses did not reveal a significant Group × Condition interaction, indicating that the magnitude of learning-related change did not differ reliably between groups. More specifically, a three-way ANOVA revealed a significant main effect of Sound, *F*_(2, 50)_ = 8.18, *p* = 0.0009, and a Group × Sound interaction, *F*_(2, 50)_ = 3.44, *p* = 0.040, indicating that velum contact patterns differed across sounds and speaker types. However, the Group × Condition interaction was not significant, *F*_(1, 50)_ = 0.10, *p* = 0.750. *Post hoc* comparisons showed that velum contact was significantly lower for nasal vowels compared to palatalized labials (*p* = 0.011, *d* = −0.56), marginally lower compared to round vowels (*p* = 0.058, *d* = 0.94), and did not differ significantly between palatalized labials and round vowels (*p* = 0.774, *d* = 1.31). These values reflect large underlying articulatory contrasts across sound types. Similar trends were observed for the palatalized labials, with bilinguals reducing velum contact while monolinguals slightly increased it. For the round vowel, the groups again diverged: bilinguals exhibited a small increase in velum touch (less nasalization), whereas monolinguals showed a slight decrease (more nasalization). While these opposing tendencies are not statistically robust, they may reflect different articulatory strategies used to approximate the novel sounds, especially in the face of uncertainty or incomplete acquisition.

#### 3.8.5 Correlations

In this section, we consider the potential link between the participants' performance on the different experimental tasks. Given the preliminary nature of the articulatory analysis, we have not identified a metric that could reliably define individual success in the production of the novel sounds, and are therefore leaving this particular relationship to future studies. For the remaining tasks, we assigned scores as follows: for phonetic and phonological learning, two scores were computed separately for the training (imitation) condition and the testing condition. The scores represented the mean accuracy (defined as successful production of the novel features in the expected environment) for both features collapsed, and we refer to them as “accent scores”. For serial memory, the whole-trial mean accuracy in the digit span task was computed for each participant. Lastly, for L2 proficiency, we used the scores obtained on the Spanish language test (which was administered to the bilingual group only).

We first conducted a two-tailed Pearson correlation analysis on the entire group of speakers, thus excluding proficiency (as only half of the participants had proficiency scores). We only found two significant correlations: a strong correlation between the accent score for training and the accent score for testing, r(20) = 0.607, *p* = 0.005, and a moderate inverse correlation between the accent score for training and digit accuracy, r(20) = −0.487, *p* = 0.03. When monolinguals and bilinguals were considered separately, we found no significant correlations for the former group, and only one significant correlation for the bilingual group: a strong correlation between the accent score for training and the accent score for testing, r(10) = 0.701, *p* = 0.024.

These correlations suggest a degree of consistency between training and retention in phonetic learning, particularly for bilingual participants, and point to a potential trade-off between resources allocated to accent learning vs. memory encoding. While only preliminary, these results highlight the value of examining cross-task relationships to better understand how bilingual experience shapes distinct but potentially interacting cognitive domains.

## 4 Discussion

In this study, we explored the impact of bilingualism on cognitive functions extending beyond the traditional focus on executive control. Specifically, we examined whether bilingual experience is associated with enhanced phonetic and phonological learning ability, articulatory skill in learning novel speech sounds, and serial memory. Our results provide a nuanced picture: (1) we replicated a bilingual advantage in phonetic and phonological learning of a novel accent of English, while at the same time raising questions about the effects of task complexity; (2) we found some evidence of higher articulatory adaptability to novel sounds for bilinguals; (3) we only found a group difference in primacy effects between high-proficiency bilinguals and monolinguals in the serial memory task for sequence length 6, but no other significant differences when we considered whole-trial accuracy and per-digit accuracy and all other sequence lengths, (4) we found significant correlations between the accent score for training and the accent score for testing, as well as between the accent score for training and the digit accuracy obtained in the digit span task, and (5) we uncovered a wide range of proficiency levels in the bilingual group despite self-reported (near)native proficiency by all participants. Overall, our findings reveal selective bilingual effects across sensorimotor domains, with stronger phonetic and articulatory performance but mixed outcomes in memory tasks.

The present study also highlights the potential of rtMRI as an innovative tool for investigating bilingual speech production. Our imaging approach provided high-resolution, dynamic articulator tracking with minimal need for manual correction. This is especially valuable for studying complex articulatory targets across groups with differing speech profiles, as it reduces subjectivity in landmark identification and facilitates more scalable analysis. As bilingualism research increasingly incorporates sensorimotor dimensions, imaging techniques like rtMRI offer a promising avenue for capturing subtle production differences that may elude more conventional acoustic or perceptual measures.

### 4.1 Phonetic and phonological learning of a new accent

Our findings can be compared to those of Spinu et al. (2023), who employed the same experimental paradigm to train participants on four different novel features (other than the diphthongization and tapping used in the current study, two additional features tested were epenthesis, whereby a vowel was inserted in between consonant clusters made up of [s] and a voiceless obstruent, e.g. “spy” was pronounced “suh-py”, and tag question intonation, with tag questions of the form “aren't you?” produced with a mid-low-high contour that departed from the typical rising (or falling) intonation in standard American English). A bilingual advantage was observed for all four features, with both the monolingual and bilingual group being least successful with the tapping feature. By contrast, diphthongization was the most successfully imitated by both groups, though did this not carryover to the testing phase. In testing, both bilinguals and monolinguals exhibited the highest accuracy with the novel intonation pattern in tag questions, leading the authors to speculate that this enhanced performance may be a consequence of the more global and salient nature of a suprasegmental phenomenon, characterized by longer duration. This may also explain why both groups were more successful with diphthongization in the current experiment, as it is generally characterized by longer duration than the tap sound, and it appears to be more perceptually salient. The greater acoustic amplitude of vowel targets (compared to consonants) likely also contributes to their salience and may have facilitated their learning.

Task complexity (Valian, 2015; Antoniou et al., 2015) also appears to play a part, as in Spinu et al. (2023) no differences were noted for the bilingual group between sentences containing two features and sentences containing all four features, whereas monolinguals displayed a marked decrease in performance for sentences with all four features. In the current experiment, only two features were present therefore the task was easier overall. This may be one of the reasons both groups performed better overall on both features, in both training (imitation) and testing, compared to Spinu et al. (2023). To sum up, while we have found similarities with earlier findings (bilinguals performed better than monolinguals on both features in training, and on the tapping feature in testing), we also found differences (both groups performed similarly on the diphthongization feature in testing).

Bilinguals' enhanced performance on this task aligns well with previous results showing that bilingual individuals often exhibit higher metalinguistic awareness. In particular, superior phonological awareness may strengthen the ability to discern and produce unfamiliar sounds and phonotactic configurations. These findings support theoretical frameworks such as MacWhinney (2005) Competition Model, which posits that bilinguals continually resolve competition between linguistic structures from both languages, resulting not only in stronger cognitive control but also in more efficient phonological encoding and rule abstraction. They also align with models of interactive activation (Marian and Spivey, 2003), which propose that bilinguals co-activate both languages during speech processing, leading to a more flexible and adaptive phonological system. Importantly, these findings reinforce the view that domain-specific mechanisms (e.g. phonological sensitivity, perceptual attunement, and articulatory flexibility) may play a central role in bilingual learning advantages, independent of, or in interaction with, more general executive functions. These models complement theories of bilingual sensorimotor integration, such as the Perceptual Assimilation Model and the Speech Learning Model, which emphasize the adaptive plasticity of bilingual speech systems.

### 4.2 Novel sound learning task

One of the main contributions of our study is the use of real-time MRI to observe how bilingual and monolingual speakers learn to produce unfamiliar speech sounds. The inclusion of real-time MRI was motivated by its ability to capture dynamic, multi-articulator configurations with minimal post-processing, making it uniquely suited for examining articulatory learning. While very preliminary, the articulatory findings suggest that bilinguals produced more of the gestures expected for successful acquisition of the novel sounds, which was reflected more generally in the ANOVA effect sizes for the differences between baseline and testing. This suggests a potential sensorimotor consequence of the bilingual experience, leading to greater adaptability in the speech motor system.

Taken together, these findings suggest that bilinguals adjusted their articulatory configurations more in their attempt to produce the unfamiliar sounds, whereas monolinguals did so to a lesser extent (except for the nasal vowel, for which adjustment of articulators other than the velum was unnecessary). For the nasal vowel, neither group achieved full nasalization, but bilinguals increased their nasalization twice as much compared to monolinguals. The latter produced most articulator changes with this vowel, for which successful production only required a decrease of velum touch. It is not clear why both speaker groups were not as successful with nasal vowels, but the fact that nasalization can also be a voice characteristic (in addition to marking linguistic contrasts) may have led listeners to believe that the model speaker had a naturally nasal voice and the novel sound they were learning would be different from baseline in other ways. Another possibility brought up by one of our reviewers is that nasalization is present and allophonic in both English and Spanish and may not be discernible as a novel item.

The posited enhanced articulatory skill in bilinguals aligns to some extent with earlier findings based on a tongue-twister task (Dugaillard and Spinu, 2019), though this comparison should be taken with caution given the very different nature of the experiental tasks. Overall, enhanced articulatory skill in bilinguals aligns with theoretical views emphasizing sensorimotor integration in speech production (Hickok et al., 2011), and the continuous interaction between sensory input and motor output during bilingual speech (Marian and Spivey, 2003). Other compatible models include Flege (1995) Speech Learning Model, which predicts that bilinguals' continuous exposure to multiple phonetic systems may facilitate the acquisition of novel sounds because their perceptual and articulatory systems remain adaptable, and the Perceptual Assimilation Model (Best, 1995) which posits more nuanced perceptual and articulatory mappings in bilinguals, allowing them to acquire novel sounds more effectively.

### 4.3 Serial memory

The serial memory task did not yield overall performance differences between speaker groups. However, a more granular analysis of serial position effects revealed group-specific patterns. High-proficiency bilinguals demonstrated significantly greater primacy effects than both intermediate bilinguals and monolinguals at sequence length 6, suggesting more robust encoding or rehearsal strategies under moderate memory load. Since primacy effects are generally linked to verbal working memory, whereas recency effects (which were not observed here) are more closely associated with auditory sensory memory (Nees, 2016), this finding may point to proficiency-related differences in working memory specifically.

At the same time, no group showed consistent advantages across all sequence lengths or accuracy metrics, and the overall trajectory of performance followed the expected decline with increasing memory load. These findings underscore the complexity of interpreting bilingual effects on memory: while high bilingual proficiency may facilitate encoding under specific conditions, it does not appear to confer a global advantage in serial recall. The variability observed among bilingual subgroups also challenges the assumption that higher L2 proficiency uniformly enhances working memory, aligning with recent calls to consider bilingualism as a gradient rather than binary trait (Antoniou et al., 2023; DeLuca et al., 2019).

Methodological factors may have further shaped performance. Our task used typed (rather than spoken) recall, introducing demands on motor planning and orthographic mapping that may have influenced accuracy, particularly for those with lower typing fluency. Additionally, although English was the dominant language for most bilinguals, we did not assess their default counting language. Since digit words in Spanish tend to be longer and less phonologically uniform than in English, conducting the task in English may have unevenly taxed bilinguals' encoding efficiency.

Individual differences unrelated to language experience likely contributed as well. Factors such as fatigue, stress, differences in workload, and reduced opportunities for test preparation (often linked to socioeconomic status) may have disproportionately affected participants' attention and effort regulation during the task (Mezzacappa, 2004; Noble et al., 2007; Best, 2010). This is consistent with prior research demonstrating that variables like sleep, physical activity, circadian rhythm, and musical expertise influence memory performance (Hahn et al., 2012; Kuula et al., 2015; Kim and Wang, 2017; Peretz and Zatorre, 2005; Zuk, 2014). It also reinforces concerns about isolating bilingualism as a sole cognitive enrichment factor in the absence of broader lifestyle data (Calvo et al., 2014; Schweizer et al., 2012; Bialystok et al., 2014).

Comparisons with previous studies highlight the potential role of sample composition. In Spinu (2023), bilinguals were primarily upper-year students at a competitive Canadian university, likely with higher SES, greater cognitive stimulation, and more test-taking experience. In contrast, Spinu et al. (2023) and the current study drew from a more socioeconomically diverse pool of community college students in New York. For instance, Spinu (2023) reported that bilinguals scored 51.1% on 8-digit sequences and 33.3% on 9-digit, while monolinguals scored 31.4% and 13.3%, respectively. In the sample tested in Spinu et al. (2023), monolinguals scored only 6% on 8-digit sequences, while bilinguals reached 42.1% (with no participants reaching the 9-digit block). If monolinguals in the current study had relatively higher SES than bilinguals, this could have masked group-level trends.

In sum, while our results do not show a robust overall group effect, the significant primacy advantage observed in high bilinguals points to nuanced effects that are modulated by proficiency, task characteristics, and individual context. Future research will benefit from an integrative experience-based framework that accounts for individual proficiency, demographic background, task features, and the many lifestyle factors that jointly shape cognitive performance.

### 4.4 Correlations

The strong positive correlation between training and testing accent scores replicates the findings of Spinu et al. (2023) and aligns with models emphasizing that bilinguals' articulatory routines remain adaptive and can reorganize through exposure (Flege, 1995), as well as memory consolidation processes underlying procedural learning (Ullman, 2001). Participants who received high accent scores during training retained and transferred their learning effectively. This suggests new phonological patterns can be established after brief exposure, though they are likely susceptible to decay if not reinforced through practice.

The moderate inverse correlation between training accent scores and digit accuracy does not indicate immediate cognitive resource competition, as the two tasks were administered sequentially. This relationship may instead reflect individual differences in cognitive functioning or long-term trade-offs in linguistic vs. numerical processing efficiency. The possibility arises that individuals who excel in phonetic learning rely more heavily on auditory and articulatory representations, whereas those who perform well in digit span tasks may have stronger domain-general working memory for numerical information. An alternative explanation is that cognitive fatigue effects may have played a role. Participants who invested greater effort in phonetic training may have had reduced efficiency in subsequent cognitive tasks, leading to lower digit accuracy. Future research could explore whether phonetic learning and digit span performance represent stable cognitive preferences.

### 4.5 Limitations

The relatively small sample size is the main limitation of our study. We would like to point out the time- and resource-intensive components of MRI studies, compounded by high operational costs, generally limiting the ability to test large participant groups. Second, enrolling participants willing to undergo MRI scanning poses challenges due to potential contraindications (e.g., implants, face jewelry, claustrophobia), and the commitment required. Despite these constraints, the sample size we employed aligns with established standards for similar studies. A recent study reveals that highly cited experimental fMRI studies have had median sample sizes of around 12 (Szucs and Ioannidis, 2020). In the speech articulation and bilingual cognition literature, our sample size aligns with or exceeds those in recent studies (Badin et al., 2024; Treutler and Sörös, 2021; Ruthven et al., 2023; Maekawa, 2023), many of which include fewer than 10 participants. Despite inherent limitations, these studies have provided valuable insights and have been successfully integrated into the field. Our study is particularly novel in its approach, as few investigations have focused on bilingual articulatory strategies using MRI. While our sample size is small, we observed articulatory differences between groups, supporting the study's validity, though we interpret them as preliminary. These findings offer a meaningful foundation for future work.

The varying Spanish proficiency within the bilingual group is also a challenge. Our median split, while methodologically common, may not have reflected a functionally meaningful proficiency threshold. It is possible that the proficiency score cutoff grouped participants in ways that do not correspond to actual differences in cognitive processing. With a small sample size, this decision may have further limited statistical power. These limitations highlight the importance of validating group boundaries with external cognitive or behavioral markers. This is a concern with any bilingual sample tested experimentally, as studies have pointed out a discrepancy between self-reports and actual proficiency scores on objective tests (Tomoschuk et al., 2019). Instead of relying solely on self-reports, which in our case indicated the population was relatively homogenous, we administered an objective proficiency task and found this was not entirely the case. Our work thus serves as an example, offering a more detailed and precise examination of the bilingual population in experiments. This aside, a number of commonalities in our participants' profiles justify treating them as a group. They all demonstrate intermediate to high proficiency in Spanish, which was typically the first language learned at home. Their connection to Spanish-speaking cultures and frequent use of both Spanish and English in various settings is a defining feature of their bilingual experience. Importantly, even with this reduced sample, we continue to see some of the previously observed advantages replicated.

Another limitation of the current study is the lack of a comprehensive analysis of participants' socioeconomic status and detailed educational background. These factors, as mentioned, could influence cognitive performance, including memory and learning, and they were not rigorously controlled here. Possible differences in other cognitive practices between groups (e.g., musical training, video gaming) could also have affected our results. In addition, any imbalance in cognitive-stimulating activities or stress/fatigue levels between groups could have influenced performance on tasks like digit span. While some of these aspects are difficult to quantify and remain inherent to experimental designs, others could be addressed in future studies, potentially through instruments like the Florida Cognitive Activities Scale (FCAS, Schinka et al., 2005), which align with findings that link cognitive activity to cognitive performance (Fritsch et al., 2005).

Finally, no formal audiometric or speech screening was conducted. This is a common limitation in behavioral language studies not conducted in labs with access to audiometric equipment and may be worth addressing in future work. Most participants were traditional college-aged students, with only one bilingual participant over the age of 50 (i.e. 53). The inclusion of a wider age range may have introduced some additional variability, which was not explicitly considered in the present analysis.

## 5 Conclusion

By integrating phonetic learning and sensorimotor mechanisms, this study is among the first to contribute toward a more comprehensive framework for understanding the cognitive consequences of bilingualism. Rather than suggesting a broad bilingual cognitive advantage, our findings highlight selective, task-specific adaptations in sensorimotor domains shaped by language experience. Bilingual participants demonstrated stronger learning of novel phonotactic patterns and articulatory configurations, indicating a potential bilingual strength in sensorimotor flexibility and speech motor learning. In contrast, we found no significant group differences in serial memory. These findings underscore the importance of considering attention, fatigue, task modality, and socioeconomic background when interpreting bilingual performance.

Our findings underscore the importance of moving beyond the search for a generalized bilingual advantage and instead examining the specific cognitive processes and contexts where language experience may lead to measurable effects. By focusing on understudied domains such as speech articulation and sensorimotor learning, we add to a growing, but still limited, body of work that seeks to clarify how bilingualism interacts with cognition in task-specific and individual-dependent ways. By integrating phonetic learning and sensorimotor mechanisms, this study is among the first to contribute toward a more comprehensive framework for understanding the cognitive consequences of bilingualism. Our results, while preliminary, suggest that bilingualism may influence specific cognitive domains, particularly those involving phonetic learning and articulatory adaptation, while showing more variable outcomes in others such as verbal memory. Importantly, our use of rtMRI provided a minimally supervised yet informative view of articulatory behavior, offering a methodologically robust way to investigate bilingual speech motor patterns. These findings support a shift away from broad claims about cognitive enhancement and toward examining which cognitive processes (and under what conditions) are shaped by language experience. Continued research with larger samples and attention to lifestyle variables will be essential in deepening this understanding.

## Data Availability

The raw data supporting the conclusions of this article will be made available by the authors, without undue reservation.

## Footnotes

^1^Working memory involves holding and manipulating information over short periods of time, cognitive flexibility allows individuals to shift perspectives and adapt to changing demands, and inhibitory control supports self-regulation by suppressing automatic responses in favor of goal-directed actions (Anderson, 2002; Diamond, 2013; Miyake and Friedman, 2012; Zelazo and Carlson, 2012).

^2^In English, [ɾ] appears allophonically between vowels as in *writer*, and [jɛ] occurs in common words like *yes*; in Spanish, [ɾ] is phonemic and contrasts with [r]; while [jɛ] is not typical, similar sequences like [je] occur in words such as *tierra* or *bien*.

^3^That said, we acknowledge that orthographic input may have introduced some degree of interference, particularly for participants needing to suppress familiar pronunciation patterns associated with standard spellings. This remains a relevant consideration for future work, and alternative designs, such as picture naming, may help reduce orthographic effects while preserving lexical control.
