# Supplementary material for: An integrative approach to bilingual cognition: preliminary insights into phonetic learning and sensorimotor adaptation
Source: Front Hum Neurosci. 2025 Jul 25;19:1549435. doi: 10.3389/fnhum.2025.1549435 (PMC12331681; doi:10.3389/fnhum.2025.1549435)
Supplement: Supplementary file 3 [file Data_Sheet_3.pdf]

Target Sounds, Articulatory Features, and Stimuli

We summarize below the target sound contrasts used in the study, along with their baseline counterparts and associated articulatory gestures. Each contrast was embedded in CVC words and designed to assess participants' ability to learn novel or altered phonetic features.

1. High Front Rounded Vowel [y] vs. High Front Unrounded Vowel [i]

Position: Word-medial
Baseline: [i] (high front unrounded vowel)
Target: [y] (high front rounded vowel)
Stimuli (baseline): *zeal, heel, steal, lean, teen, dean, seal, deal, keel, kneel, teal, keen, sheen, green, clean, seen*

Gestures of interest:

- Lip rounding (lips are spread in baseline)
- Tongue front position (both high front, may differ subtly)

2. Low Back Nasal Vowel [ɑ̃] vs. Low Back Oral Vowel [ɑ]]

Position: Word-medial
Baseline: [ɑ] (low back oral vowel)
Target: [ɑ̃]
Stimuli (baseline): *Bob, job, rob, sob, fob, cob, sop, cop, pop, hop, shop, chop, stop, drop, glob, snob*

Gestures of interest:

- Velum lowering (velum closed in baseline)
- Tongue body position (both low back; nasalized form may involve a lower tongue position)

3. Secondarily Palatalized Voiceless Labiodental Fricative [fʲ] vs. Plain [f]

Position: Word-final

Baseline: [f] (plain voiceless labiodental fricative)

Target: [f^j] (secondarily palatalized voiceless labiodental fricative)

Stimuli (baseline): *rough, tough, cough, puff, reef, chief, beef, roof, goof, woof, hoof, leaf, deaf, ref, Jeff, chef*

Gestures of interest:

- Tongue front position (higher and more fronted than baseline)
- Lips: labiodental articulation (lower lip contact with upper teeth; may be more tense or spread in the target form)
