# Supplementary material for: An integrative approach to bilingual cognition: preliminary insights into phonetic learning and sensorimotor adaptation
Source: Front Hum Neurosci. 2025 Jul 25;19:1549435. doi: 10.3389/fnhum.2025.1549435 (PMC12331681; doi:10.3389/fnhum.2025.1549435)
Supplement: Supplementary file 4 [file Data_Sheet_4.pdf]

Individual Articulatory Behavior in the Novel Sound Learning Task

The three figures presented in this section display individual two-dimensional scatterplots (in the horizontal dimension, labeled ”Fronting” and the vertical dimension, labeled ”Height”) of the articulators of interest for each of the three sounds in baseline and testing. It should be noted that the seemingly lower number of tokens for the lip measures (in particular the upper lip) is due to our method's difficulty of identifying the points of interest when the lips were closed (which was not an issue with tongue or velum measurements), resulting in higher numbers of (near-)identical values. Since for this reason many of the dots in the graphs are superimposed, the (erroneous) perception might be that they are missing. Overall, the plots are difficult to interpret due to the dispersion and overlap between conditions present, therefore the generalizations described below should be considered tentative.

Successful learning is expected to show systematic shifts along predicted articulatory dimensions. Thus, in the case of round vowels, successful learning would show the tongue tip in the same position as it was in baseline (high and front) or possibly higher and more front in cases of hyperarticulation, while the lips may be more fronted with possible raising of the upper lip and lowering of the lower lip compared to the unrounded baseline. Figure 7 shows that of the bilingual participants, Participant 6 (P6) exhibits the most pronounced changes in production from baseline to testing, with all of the articulators measured being visibly more fronted in testing. P1 mainly shows a change in the upper lip, with the tip being slightly lower and the wall slightly further back in the testing condition. Possible lowering of the tongue dorsum and lower lip wall also appears to have occurred. Lowering of the tongue dorsum and upper lip tip and wall are also produced by P7, while P8 exhibits some raising of the lower lip (tip and wall) and of the tongue tip. The least amount of change across conditions is observed with P13, with minimal lowering of the lower lip tip. In the monolingual group, Participant 10 shows the most visible changes from baseline to testing, having lowered the position of all articulators measured. P12 exhibits somewhat fronted tongue dorsum, lowered tongue tip, and a raised upper lip (tip and wall). Some backing of the tongue tip is noted for P14 and P19. P20 shows the least amount of change. As a group, bilinguals appear to produce more systematic articulatory changes involving the lips. Monolinguals

also show differences, but with greater variability and overlap.

For palatalized labial fricatives (shown in Figure 8), successful learning would be reflected in a higher and more fronted tongue tip compared to baseline, as well as more spreading of the lips, possibly resulting in raising of the upper lip and backing of the lower lip. In the bilingual group, we see a clear fronting shift in all articulators for P6. Forward movement in the tongue dorsum is also noted in P1, who also displays downward movement of the lower lip wall and upper lip tip, as well as a higher and less fronted position of the upper lip wall. P7 and P8 show more overlap between conditions overall, with some fronting of the tongue dorsum (also lowering for Participant 7). Participant 8 displays some raising of both lips (tip and wall) and of the tongue tip. The most notable change for P13 is dispersion increase in the tongue dorsum and tip. In the monolingual group, we see fronting and height changes for P10: lower positions for the tongue tip and dorsum and lower lip (tip and wall). P12 and P14 remain relatively stable across conditions. P14 shows a lowering trend for the tongue dorsum and upper lip tip. Raising of the upper lip (tip and wall) is observed in P19 and P20. The latter also produces more fronting of the tongue dorsum and raising and fronting of the tongue tip, as well as raising of the lower lip (tip and wall). In sum, bilinguals appear to display some fronting and/or raising trends of the tongue tip and dorsum. More lip movement is noted in monolinguals.

Moving on to the nasalized vowel, we would expect successful learning to result in decreased velum contact with the posterior wall and either no change in tongue body position or slightly lowered tongue body. Figure 9 reveals that of the bilinguals, P6 fronted all the articulators much like for the other sounds, while smaller differences between conditions are observed in the other participants. P1 lowers both lips while also backing the tongue dorsum. P7 and P8 show more precise articulations (less dispersion) for most articulators. P7 raises the lower lip and slightly fronts the upper lip, while backing the tongue dorsum. P8 raises both lips, fronts the upper lip slightly, and raises the tongue dorsum, while displaying more fronting of the tongue tip. The monolinguals (except P12 for whom the testing items could not be analyzed) show less dispersion in some cases, tongue tip and dorsum lowering (P10), some upper lip wall fronting (P10 and P19), and lower lip wall raising (P19 and P20).

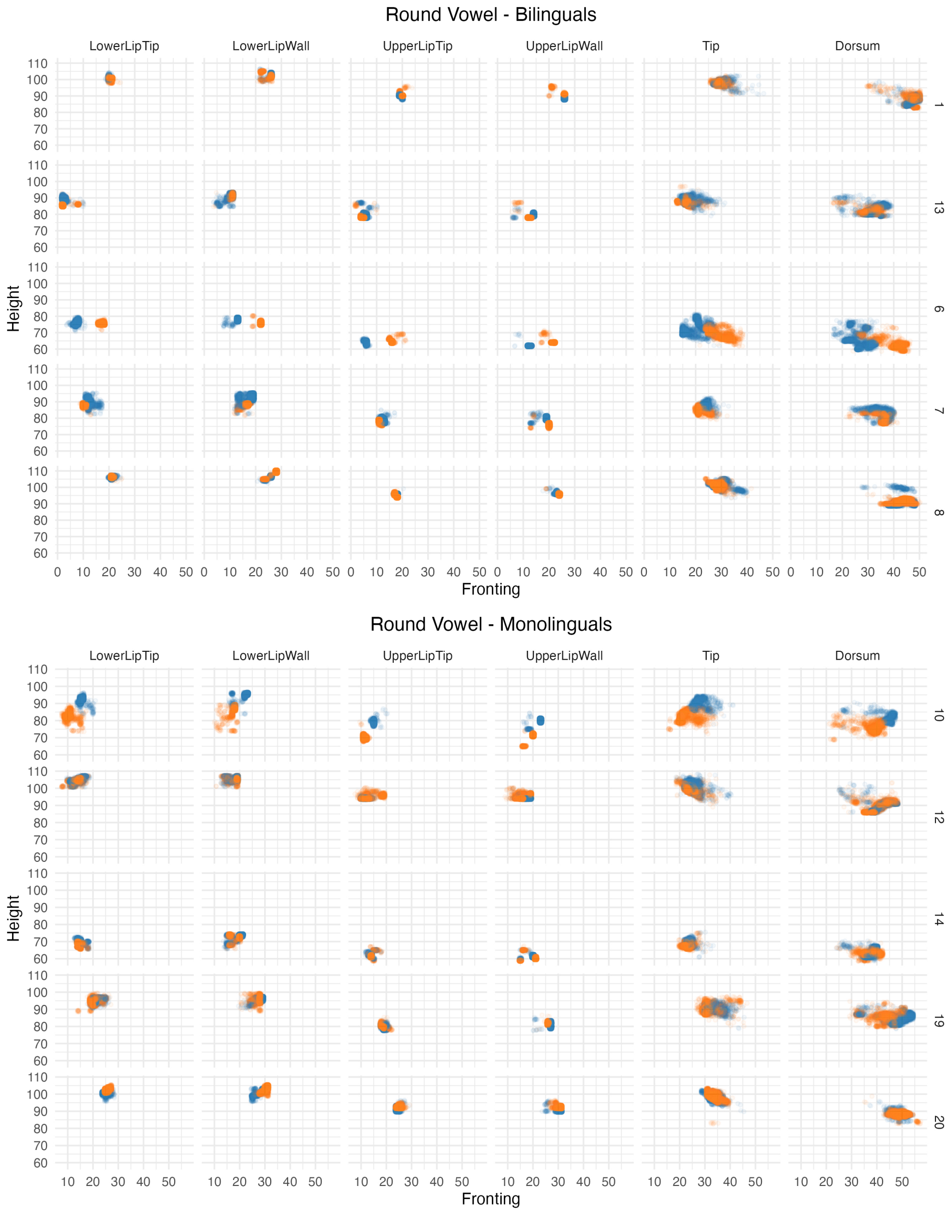

Figure 1: Scatterplots representing the horizontal and vertical position of each articulator for the round vowel, broken down by speaker. Bilingual productions are shown in the top half and monolingual productions in the bottom half. Baseline positions are shown in blue and Testing in orange. "Tip" and "Dorsum" are tongue measurements, while the remaining four measurements pertain to the lips. The higher the value, the more fronted, or raised, respectively, the articulator.

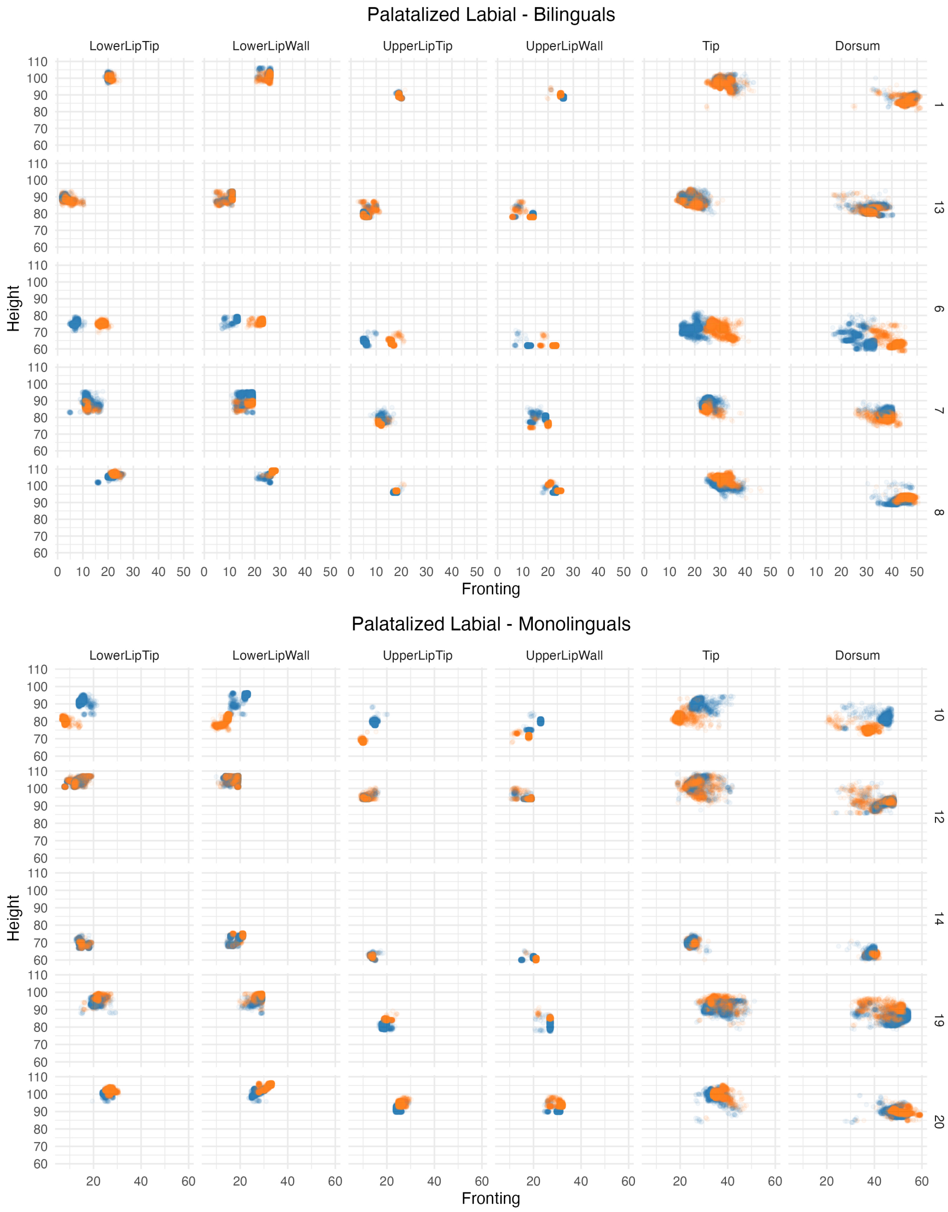

Figure 2: Scatterplots representing the horizontal and vertical position of each articulator for the palatalized labial, broken down by speaker. Bilingual productions are shown in the top half and monolingual productions in the bottom half. Baseline positions are shown in blue and Testing in orange. “Tip” and “Dorsum” are tongue measurements, while the remaining four measurements pertain to the lips. The higher the value, the more fronted, or raised, respectively, the articulator.

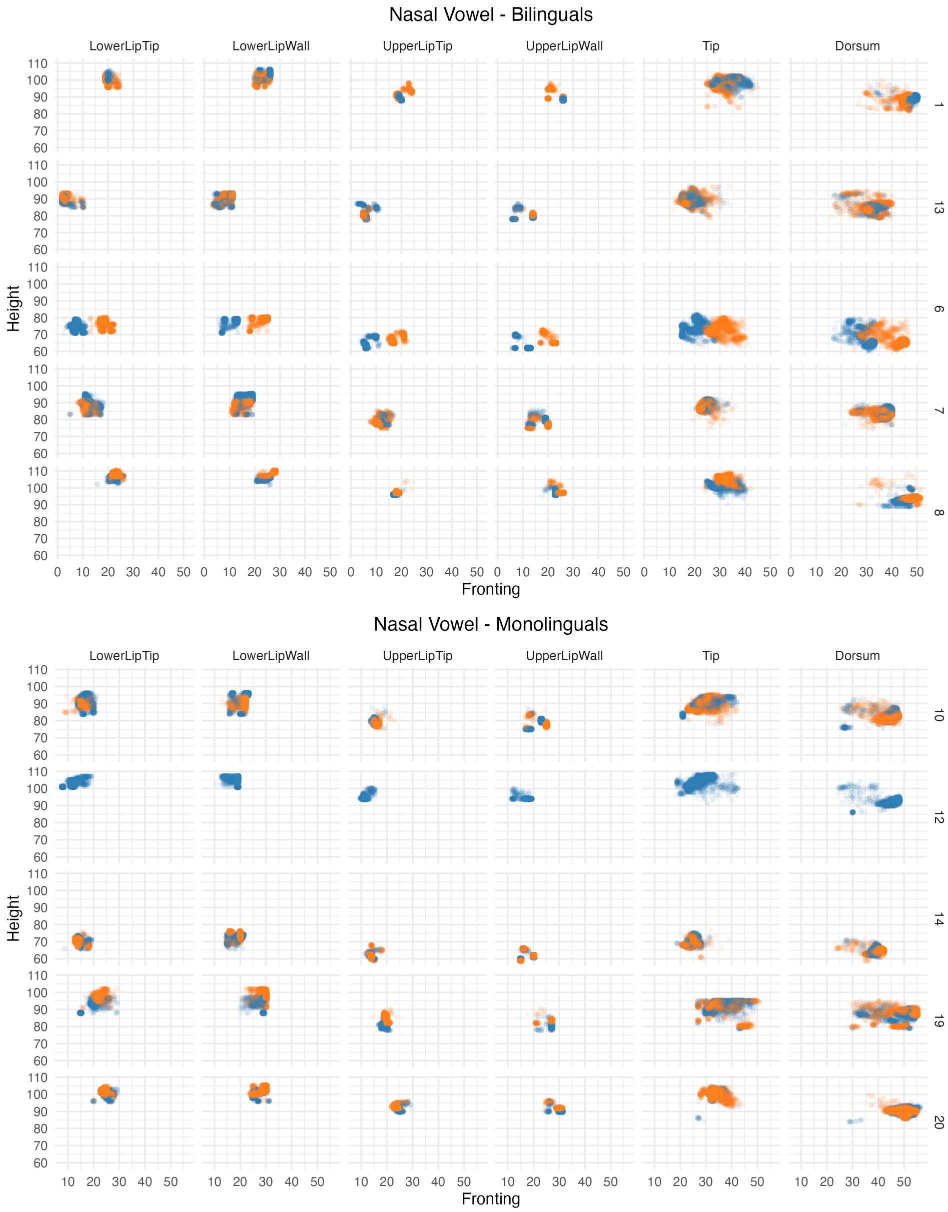

Figure 3: Scatterplots representing the horizontal and vertical position of each articulator for the nasalized vowel, broken down by speaker. Bilingual productions are shown in the top half and monolingual productions in the bottom half. Baseline positions are shown in blue and Testing in orange. "Tip" and "Dorsum" are tongue measurements, while the remaining four measurements pertain to the lips. The higher the value, the more fronted, or raised, respectively, the articulator. NOTE: Participant 12's testing data are missing due to the images obtained being too blurry.
